# Common and Rare Genetic Variants That Could Contribute to Severe Otitis Media in an Australian Aboriginal Population

**DOI:** 10.1093/cid/ciab216

**Published:** 2021-03-09

**Authors:** Sarra E Jamieson, Michaela Fakiola, Dave Tang, Elizabeth Scaman, Genevieve Syn, Richard W Francis, Harvey L Coates, Denise Anderson, Timo Lassmann, Heather J Cordell, Jenefer M Blackwell

**Affiliations:** 1 Telethon Kids Institute, The University of Western Australia, Perth, Western Australia; 2 FIRC Institute of Molecular Oncology (IFOM), Milan, Italy; 3 Faculty of Health and Medical Sciences, The University of Western Australia, Perth, Western Australia; 4 Population Health Sciences Institute, Newcastle University, Newcastle upon Tyne, UK

**Keywords:** otitis media, genetic susceptibility, *NR3C1* glucocorticoid receptor, *NREP* neuronal regeneration related protein, stereociliary bundles, cilium assembly

## Abstract

**Background:**

Our goal was to identify genetic risk factors for severe otitis media (OM) in Aboriginal Australians.

**Methods:**

Illumina^®^ Omni2.5 BeadChip and imputed data were compared between 21 children with severe OM (multiple episodes chronic suppurative OM and/or perforations or tympanic sclerosis) and 370 individuals without this phenotype, followed by FUnctional Mapping and Annotation (FUMA). Exome data filtered for common (EXaC_all ≥ 0.1) putative deleterious variants influencing protein coding (CADD-scaled scores ≥15] were used to compare 15 severe OM cases with 9 mild cases (single episode of acute OM recorded over ≥3 consecutive years). Rare (ExAC_all ≤ 0.01) such variants were filtered for those present only in severe OM. Enrichr was used to determine enrichment of genes contributing to pathways/processes relevant to OM.

**Results:**

FUMA analysis identified 2 plausible genetic risk loci for severe OM: *NR3C1* (P_imputed_1000G_ = 3.62 × 10^−6^) encoding the glucocorticoid receptor, and *NREP* (P_imputed_1000G_ = 3.67 × 10^−6^) encoding neuronal regeneration-related protein. Exome analysis showed: (i) association of severe OM with variants influencing protein coding (CADD-scaled ≥ 15) in a gene-set (*GRXCR1*, *CDH23*, *LRP2*, *FAT4*, *ARSA*, *EYA4*) enriched for Mammalian Phenotype Level 4 abnormal hair cell stereociliary bundle morphology and related phenotypes; (ii) rare variants influencing protein coding only seen in severe OM provided gene-sets enriched for “abnormal ear” (*LMNA*, *CDH23*, *LRP2*, *MYO7A*, *FGFR1*), integrin interactions, transforming growth factor signaling, and cell projection phenotypes including hair cell stereociliary bundles and cilium assembly.

**Conclusions:**

This study highlights interacting genes and pathways related to cilium structure and function that may contribute to extreme susceptibility to OM in Aboriginal Australian children.

## Teaser

Genome-wide analysis identifies common noncoding putative regulatory, as well as common and rare potentially deleterious variants directly influencing protein coding, in genes and pathways related to cilium structure and function that may contribute to severe otitis media in Aboriginal Australians.

Aboriginal Australian children have high rates of conductive hearing loss associated with otitis media (OM), which commonly occurs within 3 months of birth and progresses to severe chronic disease including chronic suppurative otitis media (CSOM) in 60% of children [1]. Otitis media involves interaction between environmental [2] and genetic [3] risk factors. Caucasians show high heritability for susceptibility to OM, increasing from 49%–71% from ages 2–4 [4]. Candidate gene and genome-wide association studies (GWAS) (reviewed [3]) identified genes/gene regions contributing to susceptibility. However, hypothesis-free genome-wide studies have not been employed for OM in Aboriginal Australians.

GWAS typically employ common variants that influence, or are in linkage disequilibrium (LD) with, single nucleotide variants (SNVs) enriched for expression quantitative trait loci (eQTLs [5]). Exome sequencing identifies putative deleterious variants influencing protein coding, as used to demonstrate that a rare variant within the middle ear-specific gene *A2ML1* (α2-macroglobulin-like 1) co-segregated with early-onset OM in an indigenous Filipino pedigree [6]. The variant lay within a founder haplotype shared by 3 unrelated otitis-prone European-American and Hispanic-American children, but was absent in non-otitis-prone children and in >60 000 exomes including the Exome Aggregation Consortium (ExAC) database [7]. De novo mutations are also thought to contribute to genetic risk as, for example, in both rare and common forms of neurodevelopmental diseases [8]. A novel mutation of large effect in an important gene can contribute to a complex disease phenotype [8].

Here we present a community-based study that uses both GWAS analysis of common variants, as well as exome analysis of common and rare variants, to identify SNVs that could contribute to genetic risk for severe OM in a Western Australian Aboriginal population.

## METHODS

### Ethical Considerations and Study Population

Study participants were from an Aboriginal community of Martu ancestry [9] in Western Australia. A memorandum of understanding with the community included permission to access hard-copy and electronic clinical records. Ethical approval was obtained from the Western Australian Aboriginal Health Ethics Committee (Reference 227 12/12). Participants (or the parent/guardian if <18 years old) signed informed consent forms to take part in the study, for access to clinical records, and to provide a DNA sample. Post-quality control (QC) variant data are lodged in the European Genome-phenome Archive (accession number EGAS00001001004). Following feedback to community, permission to publish was provided by the Board of the local Aboriginal Health Service comprising elders representing extended families residing in the area.

### Defining OM Phenotypes

Clinical records provided entries dating back >20 years. Every incidence of OM was noted for all consenting individuals, including records designated by clinical staff as acute OM (AOM), OM with effusion (OME), CSOM, perforations/healed perforations, tympanic sclerosis, and details of myringoplasty. Individuals were classified as: (i) severe OM (N = 21), based on repeated episodes including multiple entries for CSOM and/or perforations or tympanic sclerosis monitored over ≥3 consecutive years (N  = 17), or minimally 3 diagnoses of CSOM or perforations in infants <2 years old (N = 4); (ii) intermediate OM, based on at least one diagnosis of CSOM or perforations monitored over ≥3 consecutive years (N = 21); (iii) mild OM, based on a single or maximally 3 episodes of AOM (but never CSOM or perforations) monitored over ≥3 years (N = 28); and (iv) no history of any form of OM over a minimal period of 3 contiguous years (N = 41). There were 280 individuals of unknown phenotype. Supplementary Table 1 summarizes records for individuals with a definitive OM phenotype; Supplementary Table 2 provides data on age and sex. The overall OM study design is presented in Supplementary Figure 1.

### GWAS for Common Regulatory Variants

The study examined both adult metabolic diseases [10] and childhood OM. DNAs from saliva (Oragene; DNA Genotek, Ontario, Canada) were genotyped using the Illumina^®^ Omni2.5 BeadChip (Centre for Applied Genomics, Toronto, Ontario, Canada) [10]. Imputation against 1000 Genomes (1000G) haplotypes [Phase I integrated variant set release (v3)], QC procedures, and analysis of population substructure are reported elsewhere [10]. Post-QC data were available for 1 075 436 genotyped and 6 724 284 imputed SNVs for 391 individuals. This included the 21 individuals (9 males, 12 females; mean ± SD 7.29 ± 3.81 years) with a definitive severe OM phenotype. For GWAS analysis this severe OM group was compared with 370 individuals with less severe forms or no record of OM, or phenotype unknown. A case-control analysis was performed using Fisher’s Exact Test to take account of small sample size, with a genomic control to correct for relatedness. Specifically, after comparing the genotype distribution in cases and controls at each SNV using Fisher’s Exact Test, the resulting *P-*values were converted to equivalent chi-squared test statistics, the genomic control inflation factor λ was calculated as the median of the chi-squared test statistics obtained from the genotyped SNVs divided by 0.456 [11], and the raw chi-squared test statistic at each SNV was divided by λ to give the final corrected chi-squared test statistic and resulting *P-*value for that SNV. Manhattan plots were generated in R using mhtplot() in the genetic analysis package “gap.” Regional association plots were created using LocusZoom [12].

### Post-GWAS Annotation in FUMA

The FUnctional Mapping and Annotation (FUMA) Package [13] was used to characterize regions of association based on positional, eQTL, and chromatin interaction mapping. GWAS summary statistics were loaded into FUMA. SNP2GENE mapping (GWAS *P* < 10^−5^) identified independent significant SNVs based on 1000G multi-ethnic LD data. Independent significant SNVs and those in LD were annotated for gene function using ANNOVAR, regulatory functions (Regulome DB score), and 15-core chromatin state predicted by ChromHMM for 127 tissue/cell types. Effects of SNVs on gene expression were determined using eQTLs from multiple tissue/cell types from databases: eQTLgen (44 tissue types); BIOSQTL (BIO_eQTL_gene level, whole peripheral blood, 2116 healthy donors); DICE (B and T cells, monocytes, neutrophils, NK cells); and GTEx v8 (whole blood; cultured fibroblasts).

### Exome Analysis

Exome sequences were available for 72 unrelated individuals (35 pure Martu) from the post-QC GWAS sample [14]. SNV data was filtered for variants with Combined Annotation Dependent Depletion (CADD)-scaled scores ≥15 predicted to have medium (missense; splice region) to high (splice-acceptor/splice-donor; stop-gain/stop-loss; start loss) impact on protein function. Exome data included 15 unrelated children with severe OM (mean ± SD 7.86 ± 2.93 years) and 9 unrelated children (mean ± SD 13.63 ± 8.12 years) with mild OM (single episode of acute OM over ≥3 consecutive years). Extreme phenotype case-control analysis compared these 2 groups using Fisher’s Exact Test under allele-wise or dominant models and an allele-wise Trend Test for common CADD-scaled ≥ 15 variants (defined as ExAC_all ≥ 0.1). Genes with variant associations at *P* < .05 were analyzed in Enrichr [15] for enrichment of gene sets previously associated with phenotypes relevant to OM. Exome data were also filtered for rare (defined as ExAC_all ≤ 0.01) CADD-scaled ≥ 15 high/medium impact variants only ever seen in severe OM, and never in mild OM or in 48 phenotype unknown individuals. This gene list was also analyzed in Enrichr.

## RESULTS

### Characteristics of the Study Population

The 391 post-QC GWAS individuals belonged to a small number of inter-related extended pedigrees [10] (Supplementary Figure 2*A*). Principal component analysis (Supplementary Figure 2*B*) demonstrated introgression of predominantly Caucasian origin, with a tight cluster of 195 individuals of pure Martu Aboriginal ancestry.

### GWAS and Integrative post-GWAS Analyses

**Figure 1.**
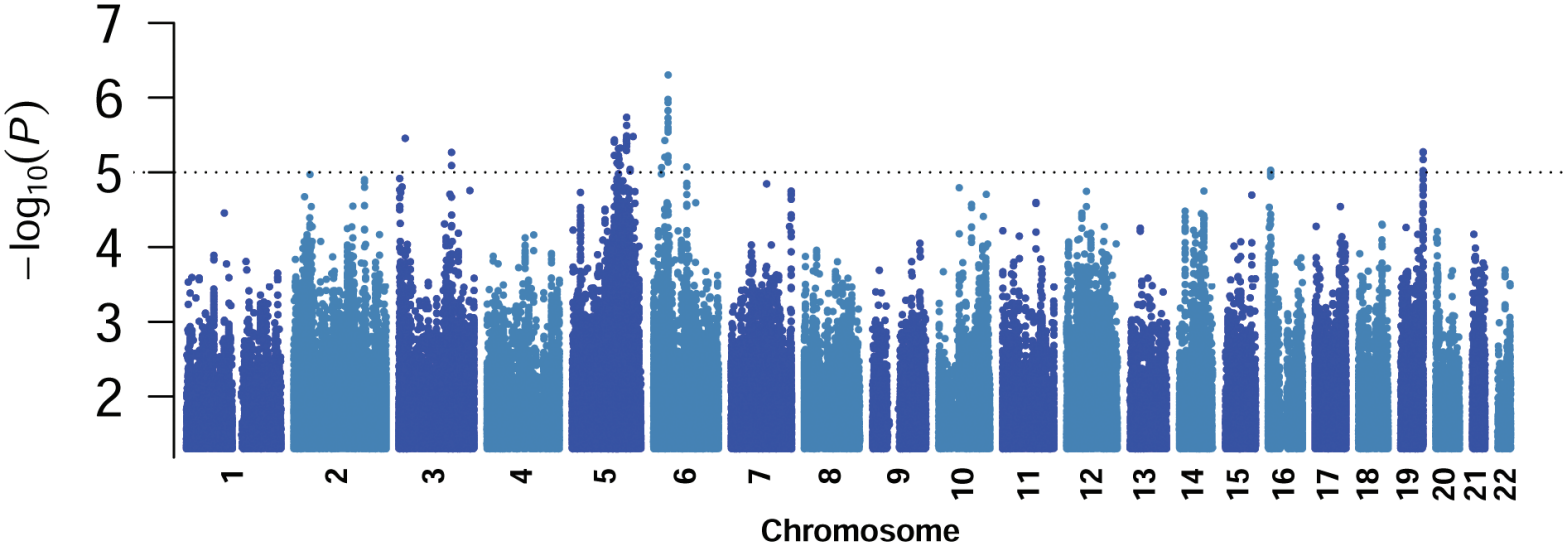
Manhattan plot of results from analysis for the 6.72M high-quality 1000G imputed SNV variants. For clarity only variants showing GWAS *P*-values < .05 are plotted. Data are for the case-control GWAS analysis undertaken using Fisher’s Exact Test with a genomic control correction to look for associations between SNVs and severe OM. The Y-axis indicates –log_10_*P* values for association, the X axis indicates the positions across each chromosome. The dotted line indicates the *P* = 1 × 10^−5^ cut-off used in the post-GWAS FUMA analysis. Abbreviations: FUMA, FUnctional Mapping and Annotation; GWAS, genome-wide association studies; SNV, single nucleotide variants.

The GWAS Manhattan plot for imputed data comparing 21 severe OM cases with 370 controls (Figure 1) showed no systematic bias (λs 0.998; Supplementary Figure 3). No hits were observed at *P* < 5 × 10^−8^. SNP2GENE identified 17 genomic loci associated with severe OM (Table 1; Supplementary Table 3). Positionally mapped SNVs mostly localized to noncoding sequence, 44% intronic, 36% intergenic, 17% intronic in noncoding RNA genes, and 3% other (including 1% exonic).

**Table 1.** Summary of SNP2GENE Results for Lead Independent Significant GWAS SNPs and Associated Gene and eQTL Information

Genomic Locus	^a^ Lead IndSigSNP Chr:bp:alleles	rsID	GWAS *P-*value	Nearest Gene(s)	Type of Gene	Distance Nearest Gene bp	Functional Location	^b^ N Pos Mapped SNVs	N eQTL SNPs	eQTL Database	^c^ eQTL Type/genes influenced
1	3:16868616: A:T	rs1866862	3.49E-06	PLCL2	Protein coding	0	intronic	41	63	BIOSQTL BIOSQTL eQTLGen GTEx/v8 eQTLCat eQTLCat eQTLCat eQTLCat eQTLCat	63 gene-level PLCL2 25 gene-level PLCL2:PLCL2-AS1 41 cis_eQTLs PLCL2 34 whole blood PLCL2 6 monocyte PLCL2 6 neutrophil PLCL2 5 neutrophil_CD15 PLCL2 5 blood PLCL2 6 fat PLCL2
2	3:138244928: C:G	rs9855074	5.38E-06	CEP70	Protein coding	0	intronic	8	7	eQTLGen BIOSQTL eQTLCat eQTLCat DICE	7 cis-EQTL FAIM 2 gene level FAIM 2 monocyte CEP70 7 LCL^d^ CEP70: FAIM 3 B cell naïve CEP70
3	5:111067185: C:CTAA	rs147647553	3.67E-06	STARD4-AS1 NREP	Antisense Protein coding	0	ncRNA_ intronic intronic	33	0	nil	nil
4	5:118132897: A:G	rs421765	7.48E-06	CTC-448D22.1	LincRNA	11742	intergenic	63	64	eQTLGen eQTLGen eQTLCat	15 cis_eQTL DMXL1 49 cis_eQTL DTWD2 3 iPSC TNFAIP8
5	5:122006325: A:G	rs13180735	4.81E-06	RP11-166A12.1 ARGFXP1	LincRNA	0	ncRNA_ intronic	6	2	eQTLCat eQTLCat eQTLgen	2 CEDAR_platelet SNX2 2 LCL^d^ PPIC 2 cis-EQTL CTB-36H16.2
6	5:123136698: C:T	rs257141	6.61E-06	KRT18P16	Pseudogene	163600	intergenic	10	0	nil	nil
7	5:124041059: C:G	rs6860814	8.10E-06	ZNF608	Protein coding	0	intronic	3	0	nil	nil
8	5:126502084: A:G	rs10076402	4.71E-06	CTB-88F18.2	Pseudogene	4142	intergenic	9	9	eQTLGen BIOSQTL eQTLCat eQTLGen	1 cis_eQTL C5orf63:MARCH3 3 gene level C5orf63:MARCH3 2 T cell C5orf63 3 cis_eQTL FBN2
9	5:142676404: G:T	rs258814	3.62E-06	NR3C1	Protein coding	0	intronic	29	24	BIOSQTL eQTLCat	24 gene level NR3C1 18 blood NR3C1
10	5:143288470: C:G	rs13170657	1.83E-06	CTB-57H20.1	Sense overlapping	80132	intergenic	62	0	nil	nil
11	5:152466290: C:T	rs11744443	9.06E-06	AC091969.1	LincRNA	0	ncRNA_ intronic	20	0	nil	nil
12	5:160326888: A:G	rs10780120	3.31E-06	RP11-109J4.1	LincRNA	31896	intergenic	57	57	eQTLCat	57 brain ATP10B
13	6:21464051: C:G	rs9460636	8.59E-06	RP11-204E9.1	LincRNA	58655	intergenic	7	0	nil	nil
14	6:38637538: A:G	rs62396381	4.95E-07	GLO1:BTBD9	Protein coding	6162	intergenic	153	130	eQTLGen eQTLGen eQTLGen eQTLGen eQTLGen eQTLGen BIOSQTL eQTLCat DICE	61 cis_eQTL GLO1 30 cis_eQTL_GLO1:ZFAND3 7 cis_eQTL GLO1:MDGA1 17 cis_eQTL MDGA1:ZFAND3 6 cis_eQTL GLO1:MDGA1:ZFAND3 1 cis_eQTL MDGA1:ZFAND3:CCDC167 3 gene level GLO1 1 monocyte naïve GLO1 4 B cell naïve RP1–153P14.8
15	6:87510924: C:T	rs4291075	8.42E-06	RP1-253B10.2	Pseudogene	32273	intergenic	8	0	nil	nil
16	16:6614413: A:G	rs2192643	9.33E-06	RP11-420N3.2 RBFOX1	Transcript Protein coding	0	ncRNA_ intronic	16	0	nil	nil
17	19:59028585: A:G	rs11545185	5.30E-06	ZBTB45	Protein coding	0	exonic	44	43	eQTLCat eQTLCat eQTLCat eQTLCat eQTLCat	^e^ 38 blood MZF1 43 monocytes CD14 MZF1 42 T cell CD4 MZF1 28 T cell CD8 MZF1 3 monocyte LPS MZF1

**Note:** More details of genomic loci are provided in Supplementary Table 3.

**Abbreviations:** ^a^ Lead IndSigSNP = GWAS top SNV; ^b^ Pos, positionally mapped SNVs for the locus from SNP2GENE analysis; ^c^ eQTL type/genes whose expression is associated with these eQTLs (note BIOSQTL = blood). ^d^ LCL, T cell leukemia cell line; ^e^ Many eQTLs associated with expression of numerous genes: ZNF584, ZNF132, CTD-2619J13.17, MZF1, AC016629.3, UBE2M, SLC27A5, CTD-2619J13.14, ZNF324, ZNF446, TRIM28, RP55, CHMP2A, ZNF544, A1BG-AS1, ZNF837, ZNF329, A1BG, MIR4754, AC010642.1, AC016629.8, CTD-2619J13.9, AC020915.1 (see Supplementary Figure 10), only those eQTLs associated with MZF1 gene expression are shown as the most plausible candidate in relation to cells relevant to OM.

**Figure 2.**
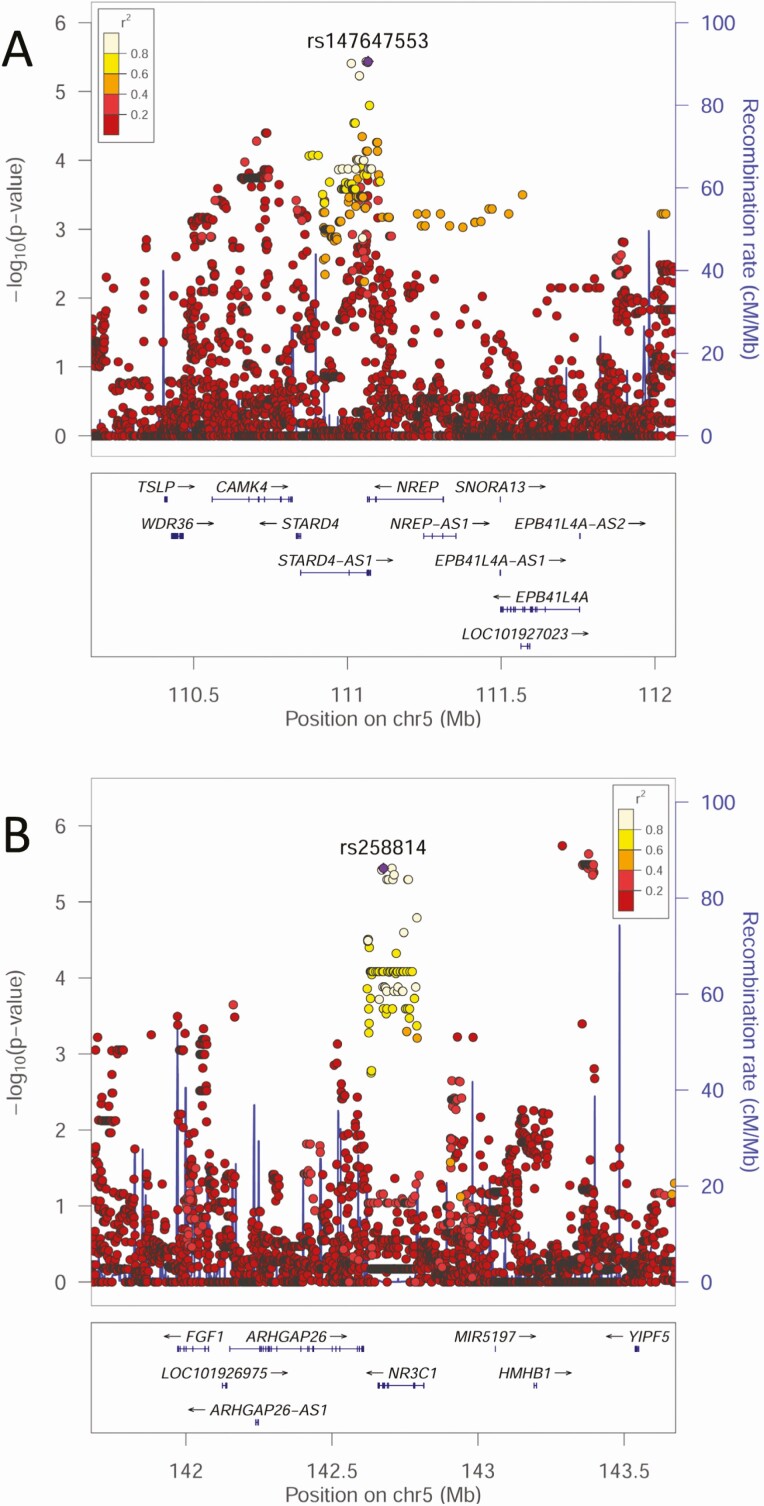
LocusZoom plots for GWAS associations identified as plausible genetic risk factors for severe OM following post-GWAS annotation: (*A*) *NREP* and (*B*) *NR3C1.* The –log_10_*P* values (left y-axis) are shown in the top section of each plot. Dots representing individual SNVs are color coded (see key) based on their population-specific LD r^2^ with the top SNV (annotated by rs ID) in the region. The right Y-axis is for recombination rate (blue line), based on HapMap data. The bottom section of each plot shows the positions of genes across the region. Abbreviations: GWAS, genome-wide association studies; LD, linkage disequilibrium; OM, otitis media; SNV, single nucleotide variants.

**Figure 3.**
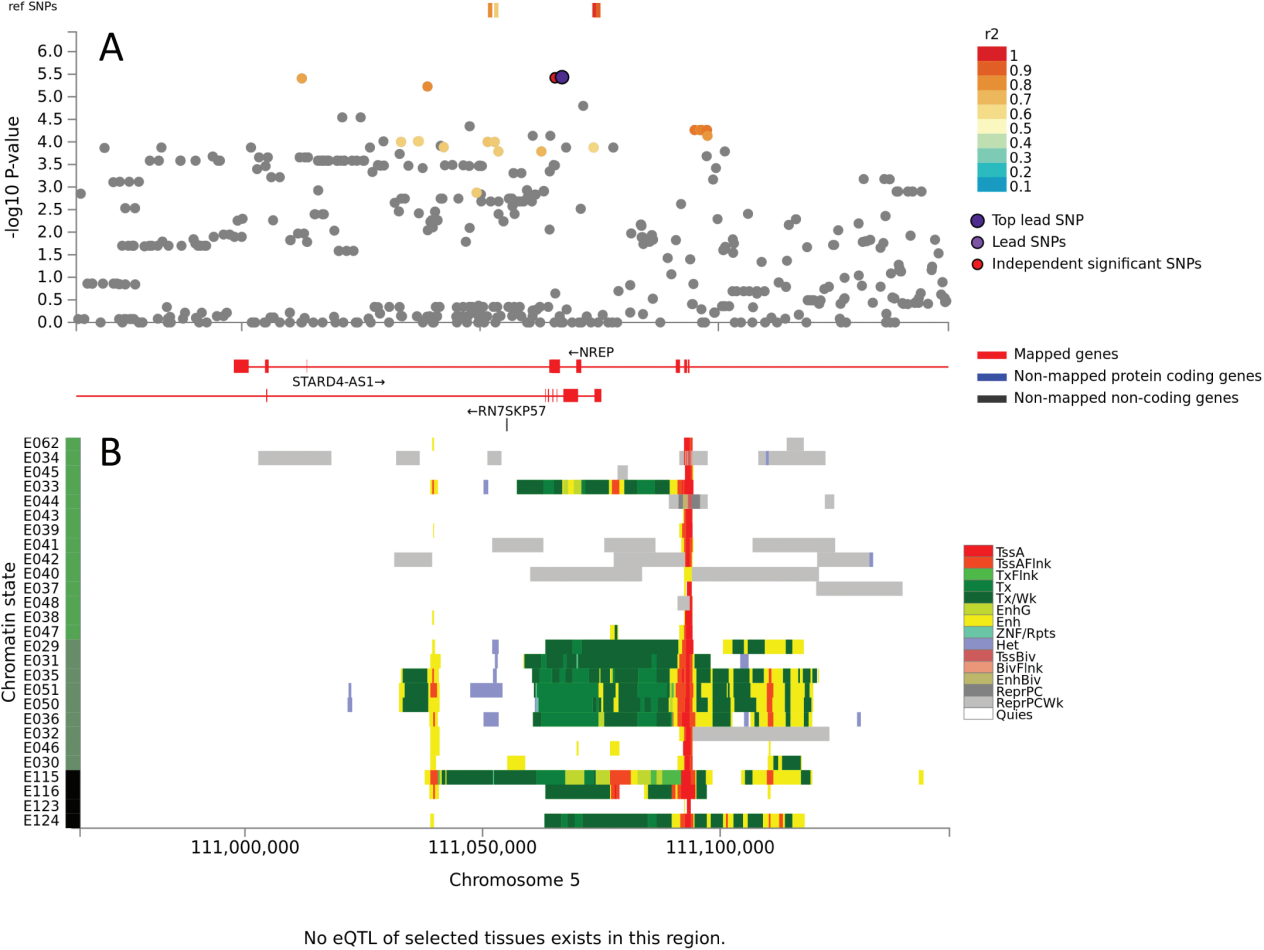
Results of positional, chromatin interaction, and eQTL activity mapping in FUMA for *NREP*. (*A*) Maps the top lead SNV, and SNVs in LD with it according to the r^2^ color-coded key, across the region of genomic locus 3. There was one additional independent significant SNV located adjacent to the top lead GWAS SNV. (*B*) Chromatin-15 states color coded for transcriptional/enhancer activity as shown in the key. Y-axis color coding relates to cell/tissue types in which chromatin interaction was mapped. There were no eQTLs mapping to genomic locus 3. Full explanation of keys provided as preamble to supplementary figures. Abbreviations: eQTL, expression quantitative trait loci; FUMA, Functional Mapping and Annotation; GWAS, genome-wide association studies; LD, linkage disequilibrium; SNV, single nucleotide variants.

No eQTLS mapped to genomic loci 3, 6, 7, 10, 11, 13, 15 and 16 (ie, there were no expression-related data to support candidacy of genes at these loci). Top SNVs at 4 of these loci (6, 10, 13, 15) were intergenic >30kb from the nearest annotated gene (Table 1). Those at loci 7, 11 and 16, were not located within genes of functional significance for OM (Supplementary Table 4). These 7 loci were not considered further. One gene with no eQTL support was *NREP* at locus 3 (GWAS *P* = 3.67 × 10^−6^, Figure 2A), despite multiple associated SNVs lying in a region of strong transcriptional activity in hematopoetic stem cells and blood (Figure 3). *NREP* encodes neuronal regeneration-related protein, variants at which associate with esophageal microbiomes [16]. Given OM association with nasopharyngeal microbiomes [17], this could represent a candidate where expression might not relate to eQTL in public domain databases.

**Figure 4.**
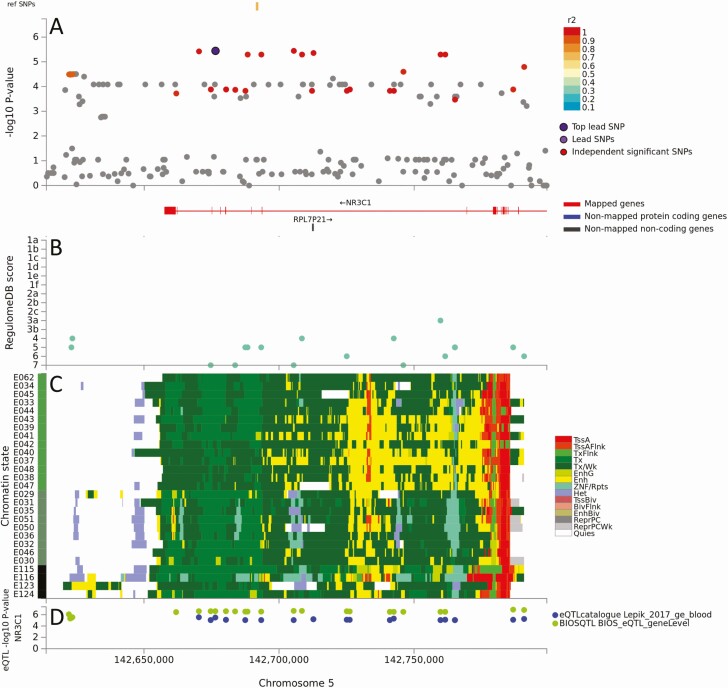
Results of positional, chromatin interaction, and eQTL activity mapping in FUMA for *NR3C1*. (*A*) Maps the top lead SNV, and SNVs in LD with it according to the r^2^ color-coded key, across genomic locus 9. There were no additional independent significant SNV. (*B*) RegulomeDB scores (Y-axis) for eQTL mapping across the locus (X-axis). (*C*) Chromatin-15 states color coded for transcriptional/enhancer activity as shown in the key. Y-axis color coding relates to cell/tissue types in which chromatin interaction was mapped. (*D*) eQTL activity for *NR3C1* (Y-axis) in different cells/tissues from public domain databases as shown in the key. Full explanation of keys provided as preamble to supplementary figures. Abbreviations: eQTL, expression quantitative trait loci; FUMA, FUnctional Mapping and Association; LD, linkage disequilibrium; SNV, single nucleotide variants.

To determine which genes in 9 loci (1, 2, 4, 5, 8, 9, 12, 14, 17) with mapped eQTLs might be risk factors for severe OM, we considered: (i) BIOSQTL gene-level (=blood) and eQTLGen cis-eQTL (=multiple tissues/cell types) support for regulation of specific genes within each locus (Table 1); (ii) eQTL-catalogue/GTEx v8/DICE support for regulation of specific genes in tissues (whole blood; brain) and cell types (immune cells) relevant to OM (Table 1); and (iii) public domain information on gene function consistent with OM pathogenesis (Supplementary Table 4). Table 1 lists eQTLs, noting that these are not always associated with expression of the gene nearest the top GWAS SNV. Of these 9 loci, we noted OM-relevant eQTL cell/tissue expression data for: (i) *PLCL2*/*PLCL2-AS1* in whole blood, monocytes, neutrophils at locus 1; (ii) *FAIM* in lymphoid cells, *CEP70* in monocytes and naive B cells, at locus 2; (iii) cis-eQTL for *DMXL1*/*DTND2* at locus 4, but no relevant cell/tissue specificity; (iv) *SNX2* in platelets, *PPIC* in lymphoid cells, at locus 5; (v) *MARCH3* in blood, C5orf63 in blood/T cells, cis-eQTL for *FBN2* but no cell/tissue specificity, at locus 8; (vi) *NR3C1* in whole blood at locus 9; (vii) *ATP10B* in brain at locus 12; (viii) cis-eQTL for multiple genes at locus 14, including *GLO1* in monocytes but no eQTL for *BTBD9*, at locus 14; and (ix) strong eQTL in immune cells for many genes, including *MZF1* (myeloid zinc finger protein), *SLC27A5* and *TRIM28*, at locus 17. Figure 4 and Supplementary Figures 4 to 11 provide detailed graphical outputs for SNP2GENE mapping. Figure 2B and Supplementary Figure 12 show parallel LocusZoom plots of GWAS mapping. Although we cannot discount roles for *PLCL2*/*PLCL2-AS1*, *FAIM, DMXL1, DTND2*, *SNX2*, *PPIC*, *MARCH3, FBN2*, *ATP10B*, *GLO1*, *MZF1*, *SCL27A5*, and *TRIM28* in OM, filtering on gene function related to pathogenesis of OM disease (Supplementary Table 4) identified *NR3C1* and *CEP70* as the most plausible genetic risk factors. *NR3C1* encodes the glucocorticoid receptor with a clear peak of association (GWAS *P* = 3.62 × 10^−6^, Figure 2B) and all eQTLs (Figure 4) chromatin-mapped to regions of strong transcriptional activity in lymphoid and myeloid cells and regulating expression (best Regulome score 3b, indicating transcription factor binding and matched transcription factor motif) only of this gene. *CEP70* shares eQTL with *FAIM* (Table 1; Supplementary Figure 5) but the lead GWAS SNV (GWAS *P* = 5.38 × 10^−6^) lies within *CEP70* (Supplementary Figures 5 and 12*B*) which is important in ciliogenesis (discussed below).

### Exome Variants Associated with Severe CSOM

**Figure 5.**
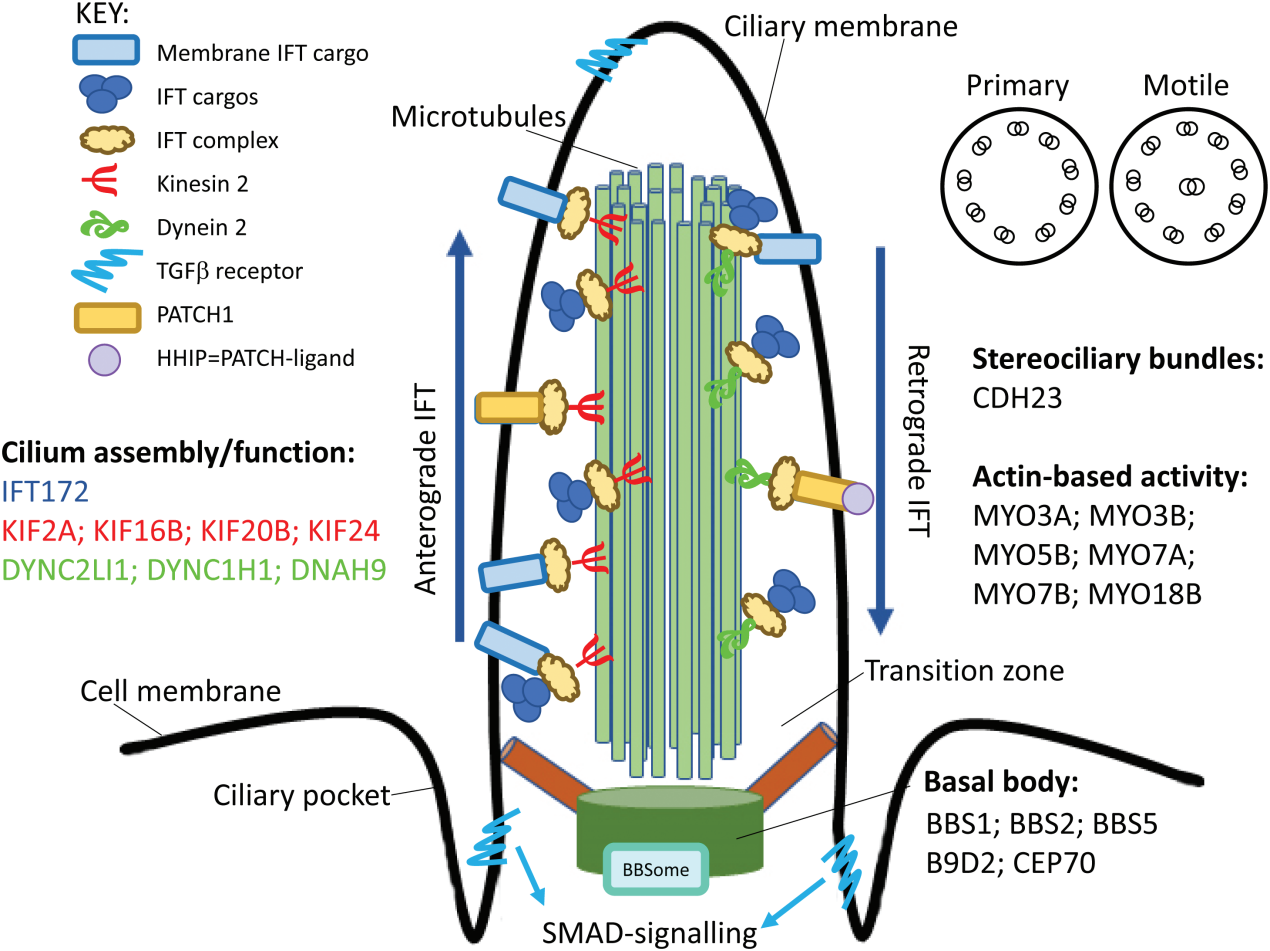
Model of cilium assembly and function highlighting some of the genes that carry putative deleterious variants (CADD-scaled score ≥15) associated with severe OM and/or where such variants were only ever observed in individuals with severe OM. Details of the full lists of genes carrying such variants are provided in Table 2 and in Supplementary Table 7. Abbreviation: OM, otitis media.

A total of 59 987 medium to high impact variants in 72 exomes were identified. Of these, 11 816 had CADD-scaled scores ≥ 15, subsets of which were used as follows. First, we filtered for 1838 common variants (ExAC_all ≥ 0.1) and carried out extreme phenotype analysis comparing 15 severe with 9 mild OM children. Associations at *P* ≤ .05 were observed for 63 variants in 61 genes (Supplementary Table 5), which were taken forward for gene set enrichment analysis (Table 2). Comparison with MGI Mammalian Phenotype Level 4, GO Biological Process 2018, Jensen Compartments and Jensen Tissues tables showed significant enrichment for genes involved in ear function (*GRXCR1*, *CDH23*, *LRP2*, *FAT4*, *ARSA*, *EYA4*, *SLC52A3*, *CTBP2*), notably with stereocilium and stereociliary bundles. Second, we filtered for rare variants (ExAC_all ≤ 0.01) only ever observed in severe OM (N = 15) and never in mild OM (N = 9) or in 48 OM phenotype unknown exomes. This filter identified 1094 variants in 1011 genes with medium (N = 1035 missense; N = 7 splice-region) to high (N = 37 stop-gain; N = 9 splice-donor; N = 4 splice-acceptor; N = 1 stop-lost; N = 1 start-lost) impact (Supplementary Table 6). The 15 severe OM exomes each carried unique variants at mean ± SD 80 ± 37 of the 1011 genes. Analysis of these 1011 genes in Enrichr showed significant (Table 3) enrichment for genes expressed in NK cells, adult lung cells, and monocytes, and for genes associated with MGI Mammalian Phenotype Level 4 “abnormal ear physiology,” GO Molecular Function 2018 ATPase activity and actin binding, NCI Nature 2016 β1- and α6β4-integrin ligand interactions, and Syndacan-1-mediated signaling, CheA 2016 tables including SMAD4 (TGFβ signaling), HNF4A (hypoxia), and CEBPD (inflammation) ChipSeq data, and with Jensen Compartments including cell projections, lamellipodium, actin-based cell projection, ciliary membrane, α7β1-integrin, cilium, and stereocilium. Supplementary Table 7 provides a complete listing of each gene-set, including genes involved in the ciliary membrane, stereociliary bundles, actin-based cell projections, and the basal body (Figure 5). The STRING diagram in Supplementary Figure 13 shows extensive interactions between the 44 genes with putative deleterious variants that overlap significantly (*P* = 1.16 × 10^−4^; P_adjusted_ = 0.011) with the 482 genes associated with “cilium” in the Jenson Compartment database.

**Table 2.** Results of Enrichr Analysis for Gene Sets Identified from 63 Putative Pathogenic Variants (CADD-scaled ≥ 15; ExAC_all ≥ 0.1) from 61 Genes Associated with Extreme OM Phenotype

Table and Term	Overlap	*P-*value	Odds Ratio	Combined Score	Genes
MGI Mammalian Phenotype Level 4					
MP:0004515 abnormal vestibular hair cell stereociliary bundle morphology	2 of 10	4.05E-04	65.57	512.18	GRXCR1; CDH23
MP:0003878 abnormal ear physiology	2 of 11	4.94E-04	59.61	453.78	CDH23; LRP2
MP:0002856 abnormal vestibular ganglion morphology	2 of 12	5.92E-04	54.64	406.11	ARSA; GRXCR1
MP:0004522 abnormal orientation of cochlear hair cell stereociliary bundles	2 of 14	8.13E-04	46.84	333.23	CDH23; FAT4
MP:0004363 stria vascularis degeneration	2 of 15	9.37E-04	43.72	304.85	GRXCR1; CDH23
MP:0002857 cochlear ganglion degeneration	3 of 64	9.85E-04	15.37	106.39	ARSA; GRXCR1; CDH23
MP:0004742 abnormal vestibular system physiology	2 of 20	0.002	32.79	209.51	GRXCR1; CDH23
MP:0004532 abnormal inner hair cell stereociliary bundle morphology	2 of 22	0.002	29.81	184.76	GRXCR1; CDH23
MP:0004491 abnormal orientation of outer hair cell stereociliary bundles	2 of 23	0.002	28.51	174.19	CDH23; FAT4
MP:0004748 increased susceptibility to age-related hearing loss	2 of 25	0.003	26.23	155.89	CDH23; LRP2
MP:0004521 abnormal cochlear hair cell stereociliary bundle morphology	2 of 28	0.003	23.42	133.91	GRXCR1; CDH23
MP:0011967 increased or absent threshold for auditory brainstem response	5 of 354	0.004	4.63	25.06	ARSA; GRXCR1; EYA4; CDH23; LRP2
MP:0004527 abnormal outer hair cell stereociliary bundle morphology	2 of 34	0.005	19.29	102.89	GRXCR1; CDH23
MP:0004736 abnormal distortion product otoacoustic emission	2 of 41	0.007	15.99	79.47	EYA4; CDH23
MP:0001967 deafness	3 of 129	0.007	7.62	37.63	ARSA; GRXCR1; CDH23
MP:0004738 abnormal auditory brainstem response	4 of 44	0.008	14.90	72.01	EYA4; CDH23
MP:0006325 impaired hearing	3 of 140	0.009	7.03	33.11	GRXCR1; CDH23; LRP2
MP:0004362 cochlear hair cell degeneration	2 of 50	0.010	13.11	60.13	GRXCR1; CDH23
MP:0000031 abnormal cochlea morphology	2 of 59	0.014	11.11	47.44	CDH23; FAT4
Go Biological Process 2018					
Sensory perception of sound (GO:0007605)	4 of 81	1.09E-04	16.19	147.68	GRXCR1; SLC52A3; CDH23; LRP2
Jensen Compartments					
Stereocilium	2 of 37	0.006	17.72	91.61	GRXCR1; CDH23
Stereocilium bundle	2 of 44	0.008	14.90	72.01	GRXCR1; CDH23
Cluster of actin-based cell projections	3 of 154	0.008	7.34	35.47	GRXCR1; CDH23; LRP2
Jensen Tissues					
Hair cell	3 of 91	0.003	10.81	63.89	GRXCR1; CTBP2; CDH23
Vestibular hair cell	2 of 27	0.003	24.29	140.62	GRXCR1; CDH23
Inner hair cell	2 of 37	0.006	17.72	91.61	CTBP2; CDH23

**Table 3.** Results of Enrichr Analysis for Gene Sets Identified from 1094 Putative Pathogenic Variants (CADD-scaled ≥ 15) ExAC_all ≤ 0.01) from 1011 Genes Found Only With Extreme OM Phenotype

Table and Term	Overlap^a^	*P-*value	Adjusted *P-*value	Odds Ratio	Combined Score
MGI Mammalian Phenotype Level 4					
MP:0003878 abnormal ear physiology	6 of 11	6.10E-06	.016	10.79	129.56
GO Molecular Function 2018					
ATPase activity (GO:0016887)	29 of 203	4.22E-07	4.86E-04	2.83	41.48
Actin binding (GO:0003779)	28 of 254	9.27E-05	.053	2.18	20.25
Jenson Compartments					
Cell projection	150 of 1774	1.52E-10	3.46E-07	1.67	37.82
Lamellipodium	26 of 168	3.44E-07	2.62E-04	3.06	45.57
Actin-based cell projection	25 of 172	1.89E-06	8.62E-04	2.88	37.90
Cell projection membrane	34 0f 284	2.88E-06	8.22E-04	2.37	30.22
Cell projection part	78 of 913	3.78E-06	9.59E-04	1.69	21.10
Ciliary membrane	15 of 79	8.35E-06	.002	3.76	43.92
Basement membrane	15 od 91	4.82E-05	.006	3.26	32.41
Integrin alpha7-beta1 complex	9 of 38	8.98E-05	.009	4.69	43.66
Cilium	44 of 482	1.16E-04	.011	1.81	16.37
Filopodium	14 of 90	1.62E-04	.012	3.08	26.86
Cluster of actin-based cell projections	17 of 134	4.29E-04	.026	2.51	19.46
Stereocilium	8 of 37	4.32E-04	.025	4.28	33.14
Ciliary part	31 of 332	7.76E-04	.039	1.85	13.23
Microtubule associated complex	17 of 142	8.39E-04	.040	2.37	16.78
NCI Nature 2016					
Beta1 integrin cell surface interactions *Homo sapiens*	12 of 66	1.02E-04	.021	3.60	33.04
Syndecan-1-mediated signaling events *Homo sapiens*	9 of 46	4.22E-04	.044	3.87	30.07
Alpha6 beta4 integrin-ligand interactions Homo sapiens	4 of 11	0.002	.067	7.19	46.27
Tissue Protein Expression From Human Proteome					
nk cells	30 of 301	3.18E-04	.010	1.97	15.88
adult lung	29 of 301	6.89E-04	.010	1.91	13.87
monocytes	26 of 301	0.005619	.056	1.71	8.85
CheA 2016					
SMAD4 21799915 ChIP-Seq A2780 Human	175 of 2464	1.38E-06	8.92E-04	1.40	18.96
FOXA2 19822575 ChIP-Seq HepG2 Human	203 of 2968	2.27E-06	7.34E-04	1.35	17.58
SMARCD1 25818293 ChIP-Seq ESCs Mouse	150 of 2119	1.08E-05	.002	1.40	16.01
DROSHA 22980978 ChIP-Seq HELA Human	45 of 456	1.48E-05	.002	1.95	21.71
HNF4A 19822575 ChIP-Seq HepG2 Human	364 of 6083	5.31E-05	.007	1.18	11.65
CEBPD 23245923 ChIP-Seq MEFs Mouse	45 of 504	1.61E-04	.017	1.77	15.42
CTNNB1 20460455 ChIP-Seq HCT116 Human	76 of 988	1.69E-04	.016	1.52	13.21
SOX9 24532713 ChIP-Seq HFSC Mouse	99 of 1384	2.70E-04	.022	1.42	11.63
AR 19668381 ChIP-Seq PC3 Human	219 of 3519	3.87E-04	.028	1.23	9.67
TCF4 23295773 ChIP-Seq U87 Human	235 of 3812	3.88E-04	.025	1.22	9.58
RUNX2 22187159 ChIP-Seq PCA Human	213 of 3423	4.79E-04	.028	1.23	9.41

^a^ Full details of the genes contributing to each gene set are provided in Supplementary Table 7.

## Discussion

Here we examine association between severe OM and common and rare variants. Noncoding regulatory variants were evaluated using post-GWAS annotation in FUMA. Putative deleterious variants influencing protein coding were identified from exome data using a CADD-scaled score cut-off ≥15. CADD scores correlate with pathogenicity [18] but there is no hard cut-off to identify pathogenic variants. CADD authors suggest a cut-off at 15, the median value for all possible canonical splice site changes and nonsynonymous variants [18]. We use CADD-scaled ≥ 15 as an indicator, not a definitive measure of variant pathogenicity. We hypothesize that carriage of multiple such variants influencing genes in functional pathways relevant to OM could contribute to genetic risk.

Whilst we acknowledge the limitations of our small cohort and that the use of probands only in the exome analysis precluded assignment of likely pathogenic variants as de novo versus inherited, 2 interesting themes emerge where functional links are plausible: (i) GWAS associations with *NR3C1* and *NREP* that suggest gene-by-environment interactions; and (ii) exome-based associations with gene-sets enriched for mammalian phenotypes/processes including abnormal ear, stereociliary bundles, cilium assembly, integrin interactions, and syndecan-1 signaling. We focus our discussion on these themes.

Glucocorticoid hormones bind to NR3C1 and regulate gene expression through transrepression of proinflammatory or transactivation of anti-inflammatory pathways [19]. The lead *NR3C1* SNV and numerous eQTLs in LD mapped to regions of strong transcriptional activity in lymphoid and myeloid cells. NR3C1 could thus act as a risk factor for severe OM through perturbation of endogenous regulation of inflammation, as seen in childhood asthma [20]. Environmental stresses also act through NR3C1-mediated endogenous corticosteroid responses. In Africa, significant correlations were observed between newborn birth weight/*NR3C1* promoter methylation and culturally relevant measures of maternal prenatal stress [21]. Adverse childhood experiences also lead to novel methylation of *NR3C1* [22]. Genetic variants modifying epigenetic signatures at *NR3C1* could contribute to effects of maternal perinatal and early childhood stress on OM, an interesting hypothesis given the early onset of severe OM in Aboriginal Australians.

Association between severe OM and *NREP* could also relate to gene-by-environment interactions. NREP (= P311) binds eukaryotic translation initiation factor 3 to promote translation of isoforms 1–3 of transforming growth factor β (TGFβ1–3) [23]. NREP promotes lung [24] and renal [25] fibrosis through stimulation of TGFβ1–3 and SMADs (homologues of “mothers against decapentaplegic, drosophila”). Murine [26] and human [27] studies demonstrate the importance of TGFβ pathways in OM. Also, distinct community types of esophageal microbiomes defined by respiratory bacteria *Streptococcus* and *Prevotella* are influenced by SNVs at *NREP* [16]. Timing of nasopharyngeal colonization with respiratory bacteria (*Moraxella catarrhalis*, *Haemophilus influenzae*, *Streptococcus pneumoniae*) predicts the onset of persistent OM in Aboriginal infants [28]. Nasopharyngeal microbial composition differs between OM-prone and nonprone children [17, 29]. Future studies could examine association between *NREP* and microbiomes of CSOM-prone Aboriginal children, complementing knowledge of rare variants at *A2ML1* and *SPINK5* and middle ear microbiomes in Filipino OM patients [30, 31].

Common regulatory variants at *CEP70* were associated with severe OM. CEP70 is a centrosomal/basal body protein that regulates ciliogenesis during zebrafish embryogenesis [32], gene depletion causing dysfunctional shortened cilia that affect ear development. Exomes from children with severe vs mild OM highlighted common (EXaC_all > 0.1) variants influencing protein coding in genes (*GRXCR1*, *CDH23*, *ARSA*, *FAT4*, *CTBP2*) previously associated with abnormal hair cell stereociliary bundles. Similarly, analysis of rare variants influencing protein coding only seen in severe OM showed significant enrichment for genes in pathways/processes involved in “abnormal ear” function (*LMNA*, *CDH23*, *LRP2*, *MYO7A*, *FGFR1*), abnormal stereociliary bundles, and cilium assembly (Figure 5). This included genes that affect assembly/function of primary and motile cilia. In addition to developmental anomalies, human primary cilia dyskinesis is associated with persisting middle ear secretion retention, suppurative infection, and chronic OM [33]. The ciliated mucosa close to the Eustachian tube contains goblet cells which secrete mucins that prevent pathogen adherence (reviewed [34]). Thus, in addition to any contribution to hearing loss (reviewed [35]) that variants in cilium assembly might have, it is likely that they contribute to increased access of bacterial pathogens to the middle ear.

Analysis of rare variants influencing protein coding also identified gene-sets previously associated with Syndecan-1 signaling (*COL16A1*, *COL4A4*, *COL6A2*, *COL12A1*, *COL4A3*, *CASK*, *COL6A3*, *HPSE*, *MET*) and β1- and/or α6β4-integrin cell surface interactions (*FGB*, *LAMA2*, *LAMA1*, *COL4A4*, *COL4A3*, *LAMA3*, *F13A1*, *LAMB1*, *THBS2*). Syndecan-1 is a cell surface proteoglycan that mediates microbial attachment/entry and elicits inflammatory responses [36]. Integrins play a role during tympanic membrane damage and repair [37], and act as cell surface receptors for TGFβ [38]. TGFβ signaling is regulated by clathrin-dependent endocytosis at the base and proximal part of cilia [39]. TGFβ receptors localize to the ciliary tip and endocytic vesicles at the ciliary base where TGFβ stimulation increases SMAD activation. Stunted primary cilia show reduced TGFβ signaling [39]. These observations provide interesting hypotheses for ways in which variants influencing TGFβ pathways and cilium assembly/function may interact to influence severe OM.

Overall, our study highlights interacting genes and pathways that may contribute to extreme susceptibility to OM in Aboriginal Australian children. While common regulatory variants identified through the GWAS appeared biased towards immune-related mechanisms, analysis of common and rare protein-coding variants were mechanistically related to cilium assembly and function.

## Supplementary Data

Supplementary materials are available at *Clinical Infectious Diseases* online. Consisting of data provided by the authors to benefit the reader, the posted materials are not copyedited and are the sole responsibility of the authors, so questions or comments should be addressed to the corresponding author.

ciab216_suppl_Supplementary_Materials

ciab216_suppl_Supplementary_Table_S1

ciab216_suppl_Supplementary_Table_S2

ciab216_suppl_Supplementary_Table_S3

ciab216_suppl_Supplementary_Table_S4

ciab216_suppl_Supplementary_Table_S5

ciab216_suppl_Supplementary_Table_S6

ciab216_suppl_Supplementary_Table_S7

## Nonstandard Abbreviations

AHS: Aboriginal Health Service
AOM: acute otitis media
CADD: Combined Annotation Dependent Depletion
CSOM: chronic suppurative otitis media
eQTL: expression quantitative trait loci
ExAC: Exome Aggregation Consortium
FUMA: FUnctional Mapping and Annotation
GWAS: genome-wide association studies
LD: linkage disequilibrium
NREP: neuronal regeneration-related protein
OM: otitis media
OME: OM with effusion
QC: quality control
SNV: single nucleotide variants
TGFβ: transforming growth factor β

## Notes

***Acknowledgments.*** We gratefully acknowledge the tremendous contribution made by the Aboriginal community, the board, and the staff of the local Aboriginal Health Service (AHS) where our study was based, and the support of local schools in the area. Without this support the study would not have been possible. We also acknowledge the generous in-kind support provided by the AHS for travel and accommodation to allow the field collection of samples used in the study, and the generosity of the Board of the AHS in allowing access to clinical records through our Memorandum of Understanding and for granting us permission to publish our findings.

***Financial support.*** The study was supported by the Australian National Health and Medical Research Council (APP634301) and the Perron Foundation. H. J. C.’s contribution to the study was supported by 2 Wellcome Trust Senior Fellowships (087436/Z/08/Z and 102858/Z/13/Z). M. F. was supported by a Raine Visiting Fellowship to the University of Western Australia. S. E. J. held a BrightSpark Foundation Research Fellowship.

***Potential conflicts of interest.*** No authors reported conflicts of interest. All authors have submitted the ICMJE Form for Disclosure of Potential Conflicts of Interest. Conflicts that the editors consider relevant to the content of the manuscript have been disclosed.

***Disclaimer.*** The study sponsor had no role in study design, data collection, data analysis, data interpretation, or writing of the report. The corresponding author had full access to all study data and had final responsibility for the decision to submit for publication.
