# Supplementary material for: Common and Rare Genetic Variants That Could Contribute to Severe Otitis Media in an Australian Aboriginal Population
Source: Clin Infect Dis. 2021 Mar 9;73(10):1860–70. doi: 10.1093/cid/ciab216 (PMC8599203; doi:10.1093/cid/ciab216)
Supplement: ciab216_suppl_Supplementary_Materials [file ciab216_suppl_supplementary_materials.pdf]

Common and rare genetic variants that could contribute to severe otitis media in an Australian Aboriginal population

Sarra E. Jamieson[1,*], Michaela Fakiola[2,*], Dave Tang[1], Elizabeth Scaman[1], Richard W. Francis[1], Harvey L. Coates[3], Denise Anderson[1], Timo Lassmann[1], Heather Cordell[4,†], Jenefer M. Blackwell[1,†]
[1]Telethon Kids Institute, The University of Western Australia, Western Australia; [2]IFOM, the FIRC Institute of Molecular Oncology, Milan, Italy; [3]Faculty of Health and Medical Sciences, The University of Western Australia, Western Australia; [4]Population Health Sciences Institute, Newcastle University, UK
[*]Contributed equally; [†]Joint senior authors
Corresponding author: Jenefer M. Blackwell (jenefer.blackwell@telethonkids.org.au); telephone +61 400 396082; ORCID ID: 0000/0002-0784-7277

Supplementary Figures

Preamble: Information from the summary of genome-wide SNP2GENE results obtained using FUMA are provided here along with keys to aid in interpretation of main Figures 3 and 4, and Supplementary Figures S4 to S11.

Summary of SNPs and mapped genes:

#Genomic risk loci	17
#Lead SNPs	17
#Ind. Sig. SNPs	24
#Candidate SNPs	569
#Candidate GWAS tagged SNPs	461
#Mapped genes	64

Functional consequences of SNPs on genes:

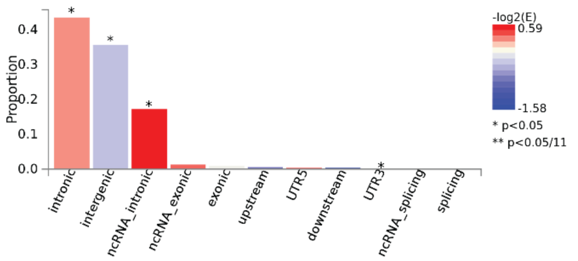

Summary per genomic risk locus:

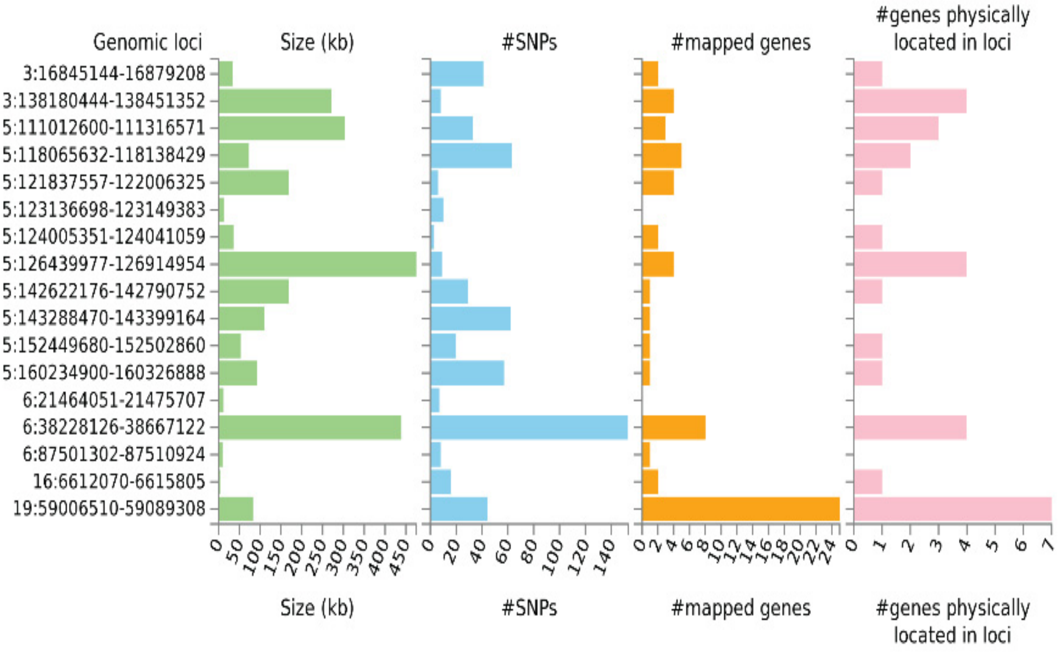

A. Positional mapping:

Shown in Parts A of each of main Figures 3 and 4, and Supplementary Figures 3 to 10.

Red: Mapped genes. Genes mapped by positional mapping are always colored red.
Blue: Non-mapped protein-coding genes.
Dark grey: Non-mapped non-coding genes.
GWAS P-value: SNVs (also known as SNPs) which are not in LD of any of significant independent lead SNPs in the selected region are colored grey.

B. eQTL mapped with respect to Regulome DB scores

Shown in Supplementary Figures S3, S5, S8, S9, S10.

The scoring scheme refers to the following available datatypes for a single coordinate.

Score	Supporting data
1a	eQTL + TF binding + matched TF motif + matched DNase Footprint + DNase peak
1b	eQTL + TF binding + any motif + DNase Footprint + DNase peak
1c	eQTL + TF binding + matched TF motif + DNase peak
1d	eQTL + TF binding + any motif + DNase peak
1e	eQTL + TF binding + matched TF motif
1f	eQTL + TF binding / DNase peak
2a	TF binding + matched TF motif + matched DNase Footprint + DNase peak
2b	TF binding + any motif + DNase Footprint + DNase peak
2c	TF binding + matched TF motif + DNase peak
3a	TF binding + any motif + DNase peak
3b	TF binding + matched TF motif
4	TF binding + DNase peak
5	TF binding or DNase peak
6	other

Note: Regulome score mapping is only shown in Supplementary Figures 3 to 10 where scores are better than 3b.

C. ROADMAP Chromatin States in 15-state models:

https://egg2.wustl.edu/roadmap/web_portal/chr_state_learning.html

Shown in Parts B of each of main Figures 3 and 4, and Supplementary Figures 3 to 10.

127 epigenomes, 5 chromatin marks

- H3K4me3
- H3K4me1
- H3K36me3
- H3K27me3
- H3K9me3

15-core chromatin state data for Blood and Skin – Y-axis* colour codes for epigenomes used in chromatin state mapping.

Epigenome ID

EID	Color	Group	Anatomy	Standerdized epigenome name
E062	#55A354	Blood & T-cell	BLOOD	Primary mononuclear cells from peripheral blood
E034	#55A354	Blood & T-cell	BLOOD	Primary T cells from peripheral blood
E045	#55A354	Blood & T-cell	BLOOD	Primary T cells effector/memory enriched from peripheral blood
E033	#55A354	Blood & T-cell	BLOOD	Primary T cells from cord blood
E044	#55A354	Blood & T-cell	BLOOD	Primary T regulatory cells from peripheral blood
E043	#55A354	Blood & T-cell	BLOOD	Primary T helper cells from peripheral blood
E039	#55A354	Blood & T-cell	BLOOD	Primary T helper naive cells from peripheral blood
E041	#55A354	Blood & T-cell	BLOOD	Primary T helper cells PMA-I stimulated
E042	#55A354	Blood & T-cell	BLOOD	Primary T helper 17 cells PMA-I stimulated
E040	#55A354	Blood & T-cell	BLOOD	Primary T helper memory cells from peripheral blood 1
E037	#55A354	Blood & T-cell	BLOOD	Primary T helper memory cells from peripheral blood 2
E048	#55A354	Blood & T-cell	BLOOD	Primary T CD8+ memory cells from peripheral blood
E038	#55A354	Blood & T-cell	BLOOD	Primary T helper naive cells from peripheral blood
E047	#55A354	Blood & T-cell	BLOOD	Primary T CD8+ naive cells from peripheral blood
E029	#678C69	HSC & B-cell	BLOOD	Primary monocytes from peripheral blood
E031	#678C69	HSC & B-cell	BLOOD	Primary B cells from cord blood
E035	#678C69	HSC & B-cell	BLOOD	Primary hematopoietic stem cells
E051	#678C69	HSC & B-cell	BLOOD	Primary hematopoietic stem cells G-CSF-mobilized Male
E050	#678C69	HSC & B-cell	BLOOD	Primary hematopoietic stem cells G-CSF-mobilized Female
E036	#678C69	HSC & B-cell	BLOOD	Primary hematopoietic stem cells short term culture
E032	#678C69	HSC & B-cell	BLOOD	Primary B cells from peripheral blood
E046	#678C69	HSC & B-cell	BLOOD	Primary Natural Killer cells from peripheral blood
E030	#678C69	HSC & B-cell	BLOOD	Primary neutrophils from peripheral blood
E115	#000000	ENCODE2012	BLOOD	Dnd41 TCell Leukemia Cell Line
E116	#000000	ENCODE2012	BLOOD	GM12878 Lymphoblastoid Cells
E123	#000000	ENCODE2012	BLOOD	K562 Leukemia Cells
E124	#000000	ENCODE2012	BLOOD	Monocytes-CD14+ RO01746 Primary Cells

*The order of the cell types is same as this legend table.

Key to colour coded regions on X-axis:

- Active TSS
- Flanking Active TSS
- Transcr. at gene 5' and 3'
- Strong transcription
- Weak transcription
- Genic enhancers
- Enhancers
- ZNF genes & repeats
- Heterochromatin
- Bivalent/Poised TSS
- Flanking Bivalent TSS/Enh
- Bivalent Enhancer
- Repressed PolyComb
- Weak Repressed PolyComb
- Quiescent/Low

D. eQTLs

Regulome scores for eQTL are shown in parts B of each of main Figure 4, and Supplementary Figures S4, S6, S9, S10 and S11. The Regulome score plot was only included where there was evidence for a direct role of the eQTL in transcription (i.e. Regulome scores ≤3b).

Color coding for gene-specific plots for eQTLs for different databases shown in the graph key (Figure 4D; Supplementary Figures S4D, S5C, S6D, S7C, S8C, S9D, S10D and S11D) is assigned arbitrarily. All eQTLs with user defined P-value threshold and tissue types are displayed.

Supplementary Figure 1

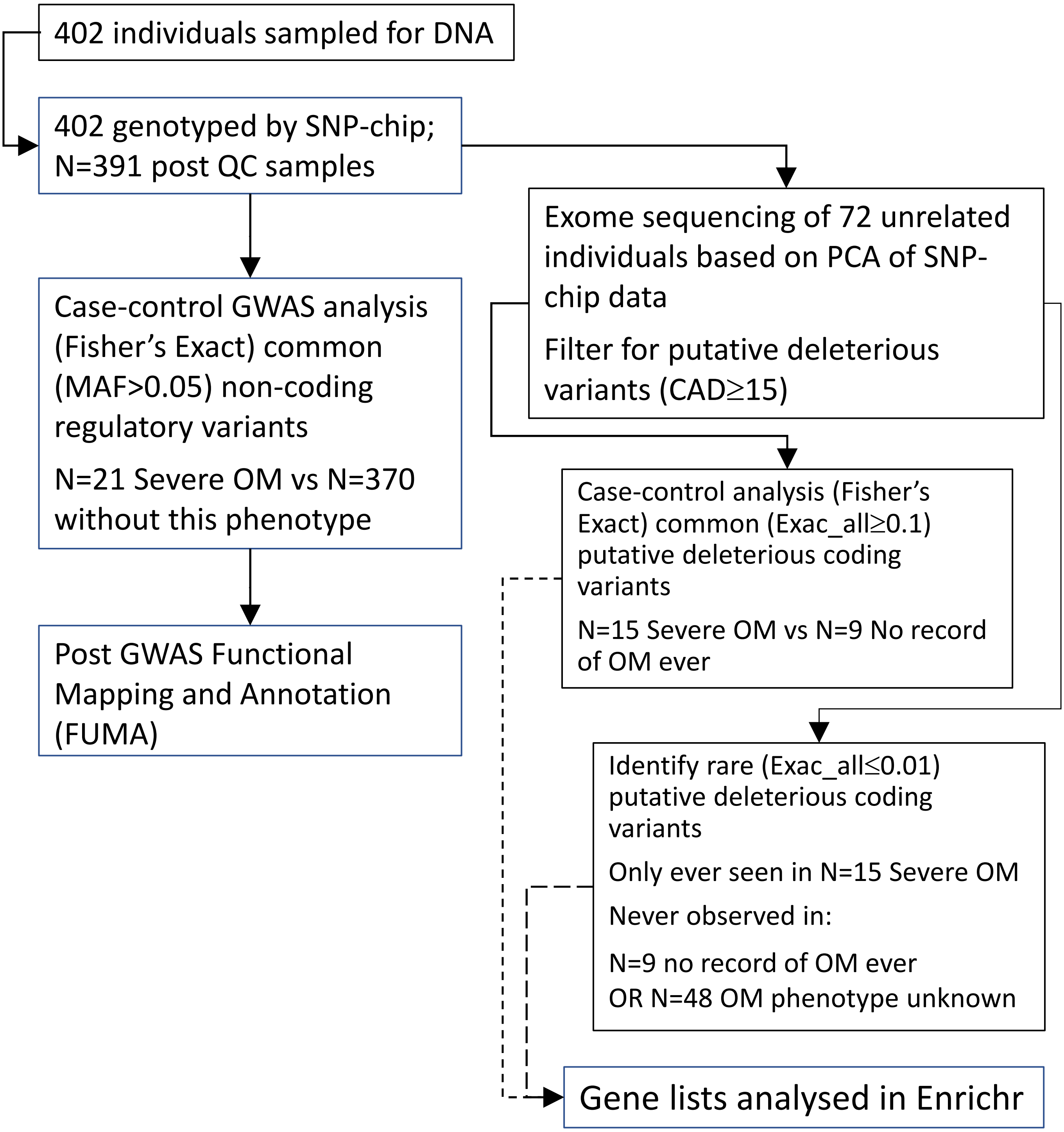

Figure S1. Diagrammatic representation of the study design.

Supplementary Figure 2

A

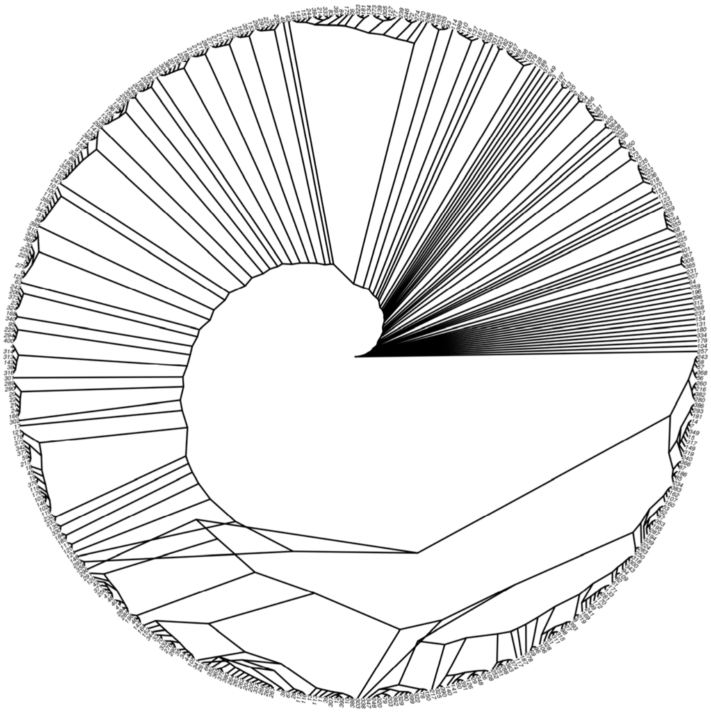

B

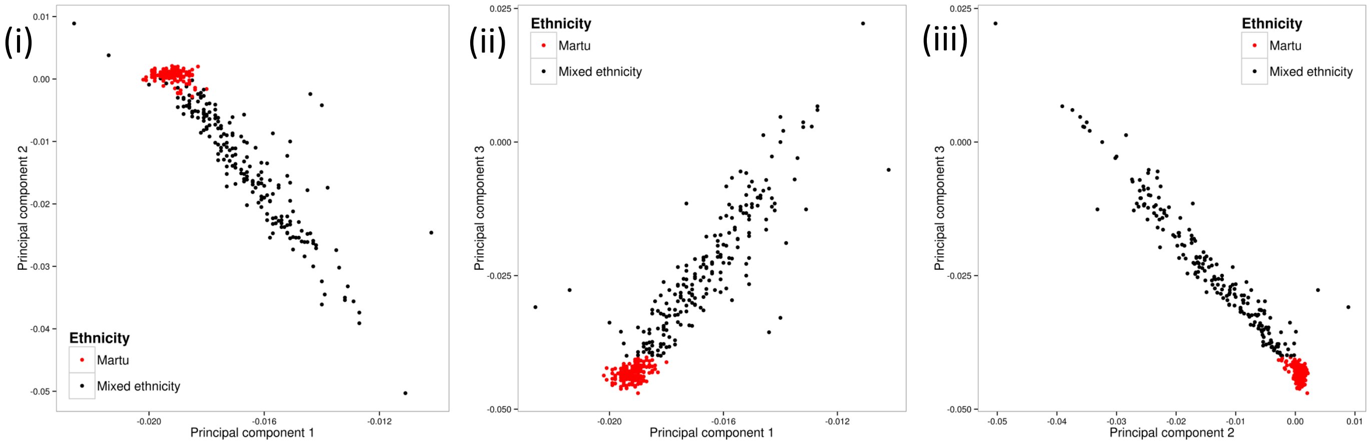

Figure S2. (A) Radial plot showing hierarchical clustering of estimated pairwise identity-by-descent allele-sharing for the 402 genotyped individuals used in the original GWAS*. The genomic kinship matrix was first calculated in GenABEL v1.7-6, and converted to a distance matrix and hierarchical cluster analysis using single linkage on the dissimilarities. The ape package was used to produce radial tree plot. (B) Principal component (PC) analysis (PCA) plots showing population substructure in the study population. A subset of 70,420 genotyped SNPs with pairwise linkage disequilibrium (LD; r2) ≤0.3 and MAF >0.01 was used in PCA (SMARTPCA within EIGENSOFT) to look at population substructure across the 402 genotyped family members. PCA plots (i) PC1 x PC2, (ii) PC1xPC3, and (iii) PC2xPC3 show individuals, color coded by ancestry (see key). Reference HapMap populations are not included at the specific request of the Board of the Aboriginal Health Service. Adapted from Figures S1 and S2 of *Anderson et al., 2015, PLOS ONE, 10(3):e0119333.

Supplementary Figure 3

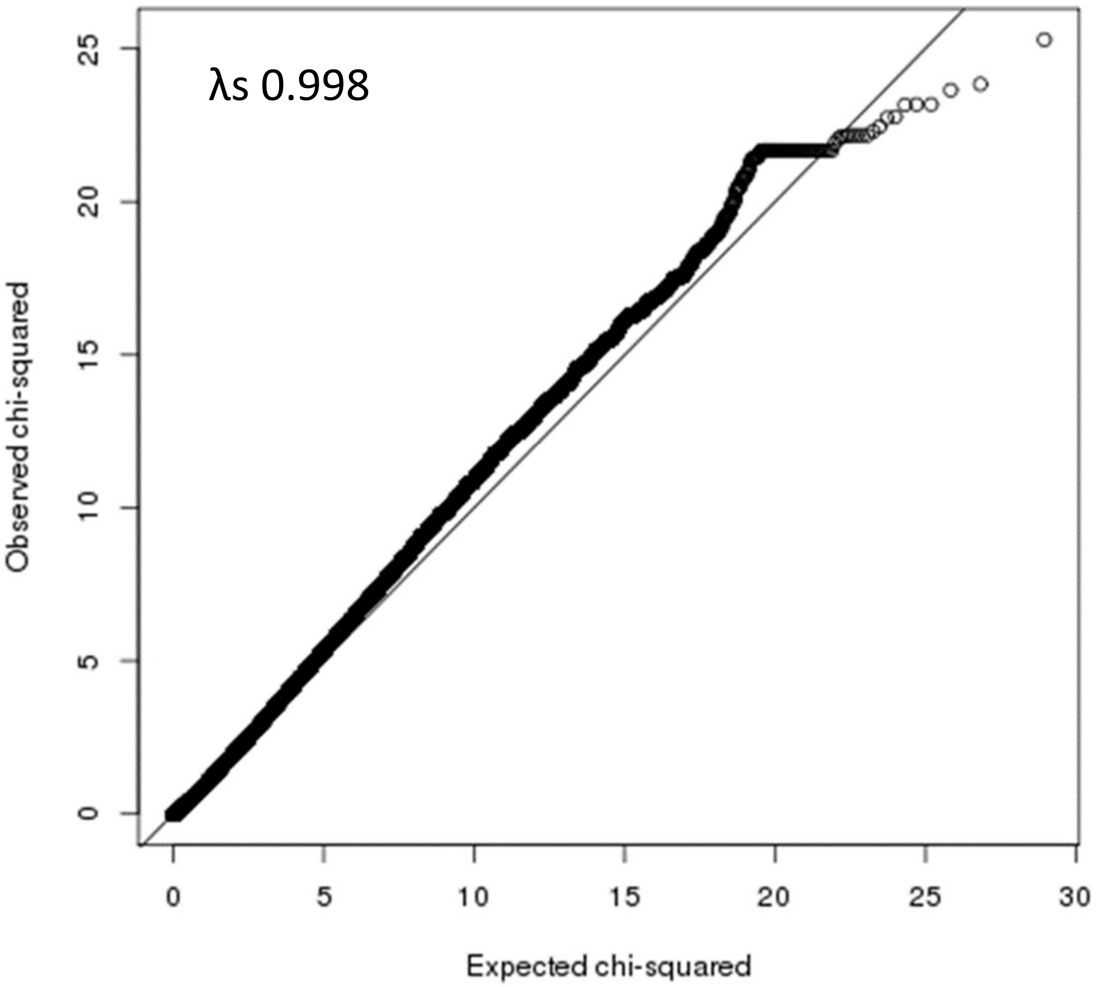

Figure S3. Quantile-quantile (Q-Q) plot P-values for imputed data for the GWAS comparing 42 CSOM *per se* cases with 67 mild/no OM controls .

Supplementary Figure 4

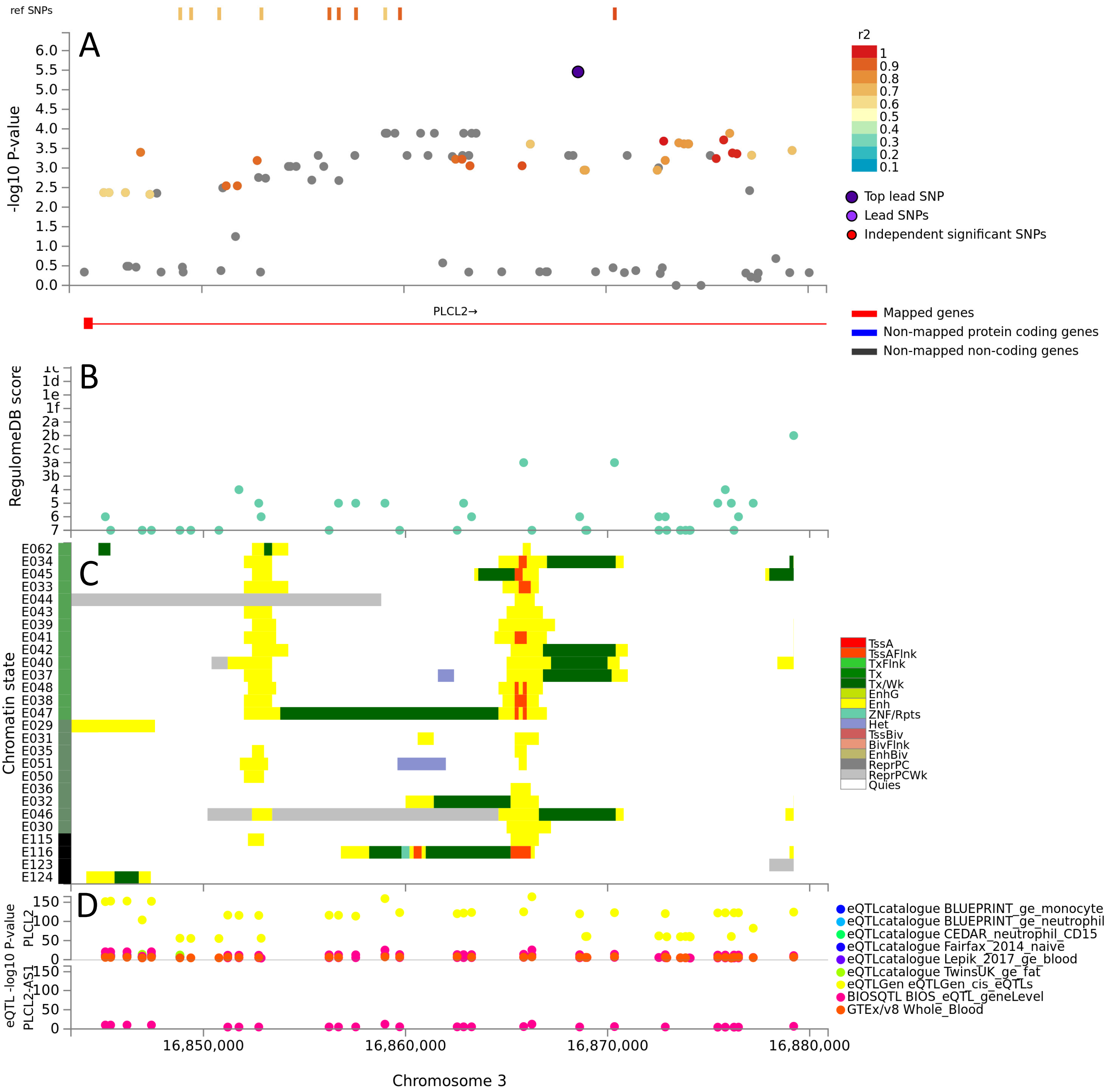

Figure S4. Results of positional, Regulome CB, chromatin interaction, and eQTL activity mapping in FUMA for genomic locus 1 (*PLCL2)*. (A) Maps the top lead SNV, and SNVs in LD with it according to the r^2 colour-coded key, across the three genes. (B) Maps eQTL according to Regulome scores. (C) Chromatin-15 states colour coded for transcriptional/enhancer activity as shown in the key. Y-axis colour coding relates to cell/tissue types in which chromatin interaction was mapped. (D) eQTL activity for *PLCL2* and *PLCL2-AS1* genes (Y-axis) in different cells/tissues from public domain databases as shown in the key. Full explanation of keys provided on introductory page for supplementary figures.

Supplementary Figure 5

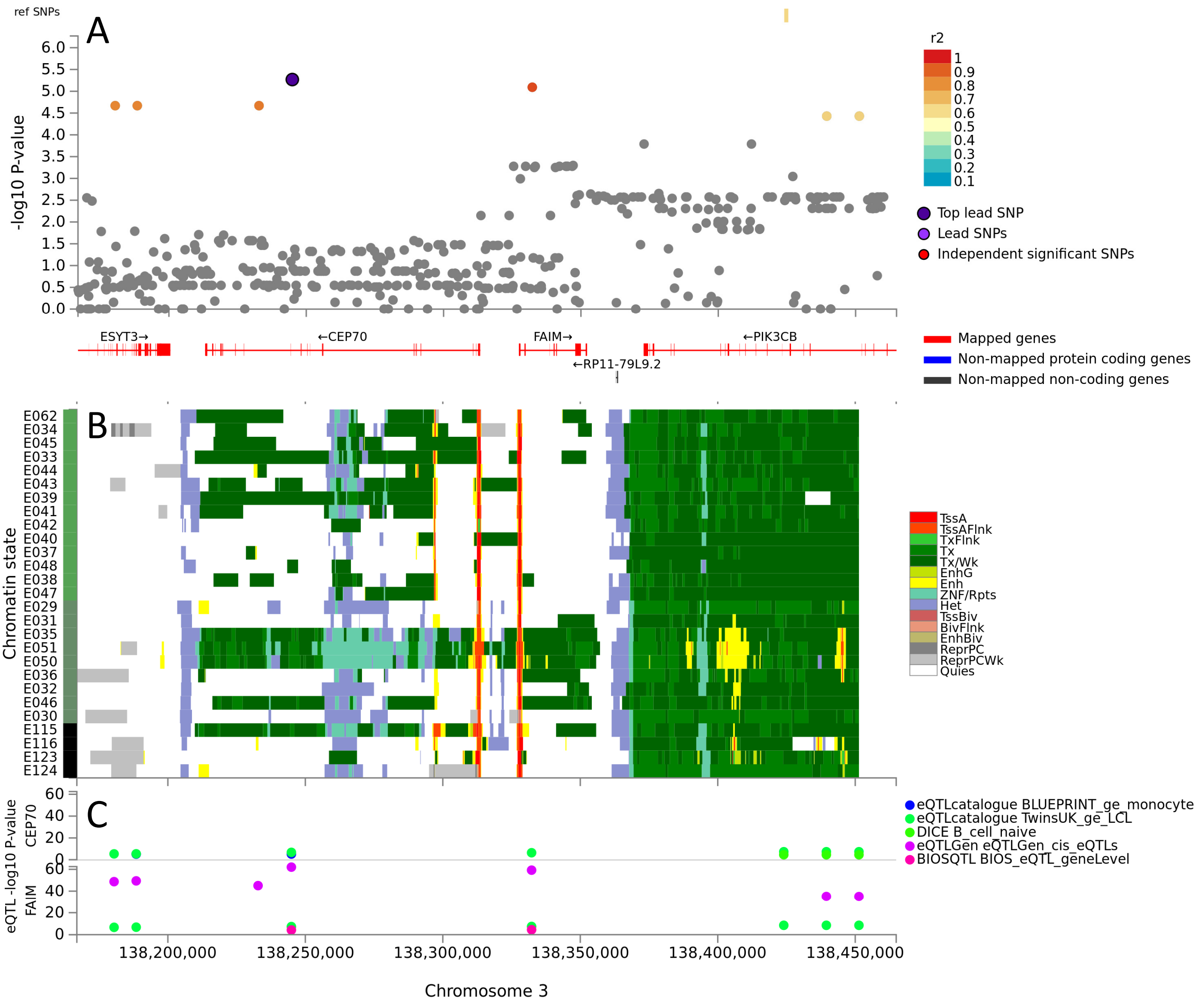

Figure S5. Results of positional, chromatin interaction, and eQTL activity mapping in FUMA for genomic locus 2 where *CEP70*/*FAIM*/*PIK3CB* share a common lead SNV. (A) Maps the top lead SNV, and SNVs in LD with it according to the r^2 colour-coded key, across the three genes. There were no additional independent significant SNVs. (B) Chromatin-15 states colour coded for transcriptional/enhancer activity as shown in the key. Y-axis colour coding relates to cell/tissue types in which chromatin interaction was mapped. (C) eQTL activity for *FAIM* and *CEP70* genes (Y-axis) in different cells/tissues from public domain databases as shown in the key. Full explanation of keys provided on introductory page for supplementary figures.

Supplementary Figure 6

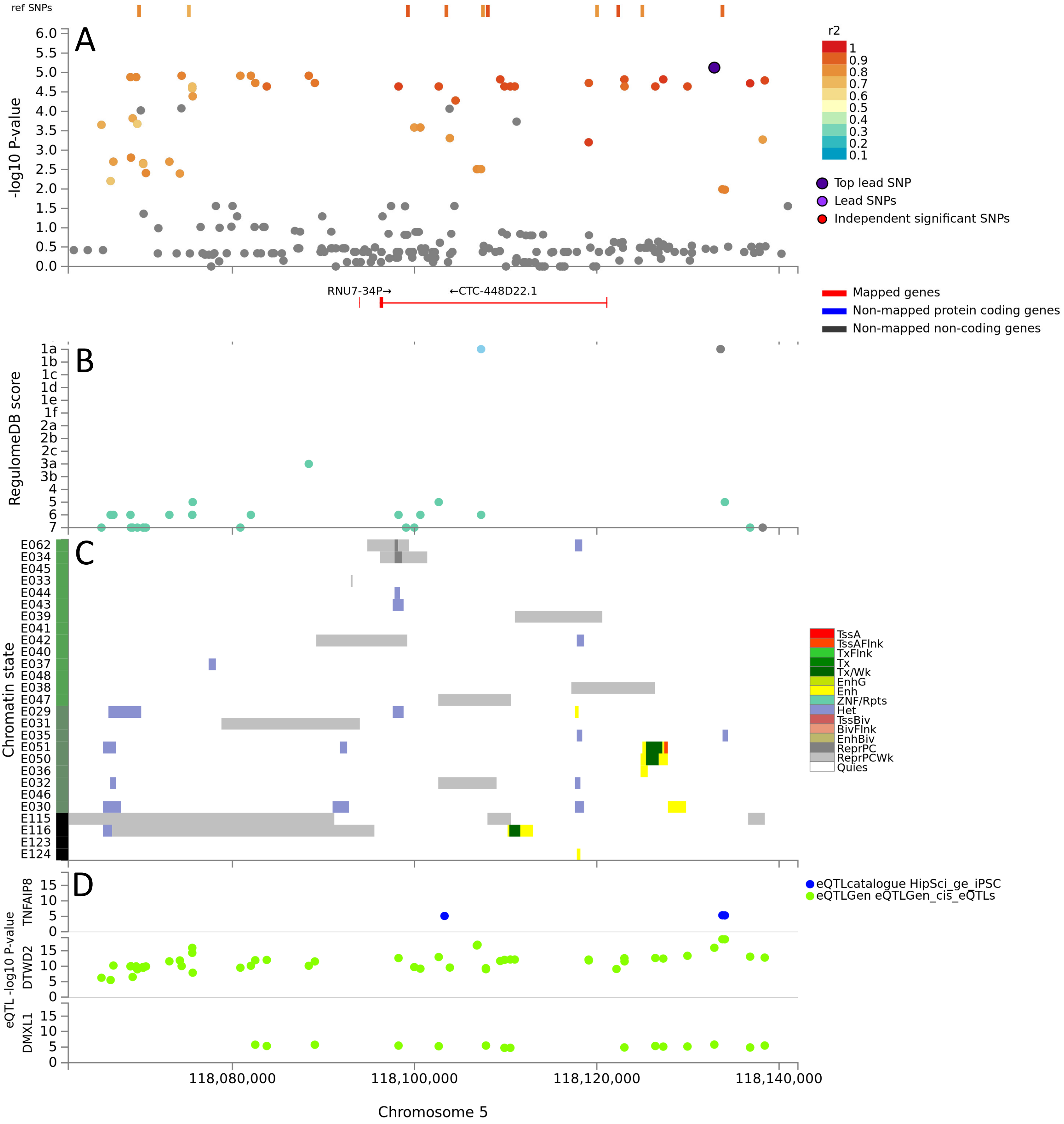

Figure S6. Results of positional, Regulome DB, chromatin interaction, and eQTL activity mapping in FUMA for genomic locus 4 (*CTC-448D22.1*). (A) Maps the top lead SNV, and SNVs in LD with it according to the r^2 colour-coded key, across the three genes. There were no additional independent significant SNVs. (B) Maps eQTL according to Regulome scores. (C) Chromatin-15 states colour coded for transcriptional/enhancer activity as shown in the key. Y-axis colour coding relates to cell/tissue types in which chromatin interaction was mapped. (D) eQTL activity for *DMXL1*/*DTWD2*/*TNFAIP3* genes (Y-axis) in different cells/tissues from public domain databases as shown in the key. Full explanation of keys provided on introductory page for supplementary figures.

Supplementary Figure 7

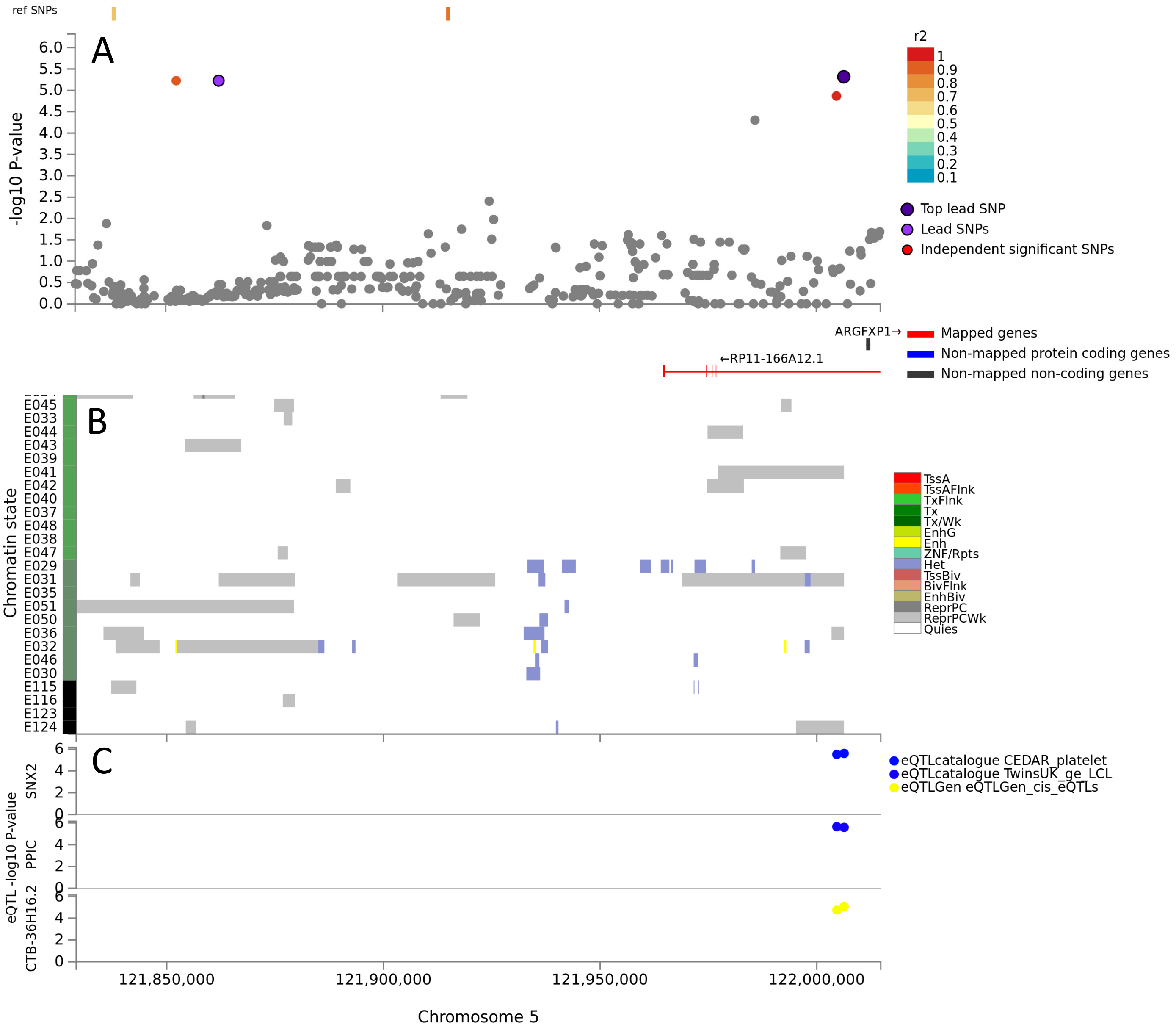

Figure S7. Results of positional, chromatin interaction, and eQTL activity mapping in FUMA for genomic locus 5 where *RP11-166A12.1/ARGFXP1* share a common lead SNV. (A) Maps the top lead SNV, and SNVs in LD with it according to the r^2 colour-coded key, across the three genes. There is one additional independent significant SNV. (B) Chromatin-15 states colour coded for transcriptional/enhancer activity as shown in the key. Y-axis colour coding relates to cell/tissue types in which chromatin interaction was mapped. (C) eQTL activity round the lead SNV for *SNX2/PPIC/CTB36H16.2* genes (Y-axis) in different cells/tissues from public domain databases as shown in the key. These gene lie immediately distal to the pseudogene *ARGFXP1* on chromosome 5q (see Figure S11). Full explanation of keys provided on introductory page for supplementary figures.

Supplementary Figure 8

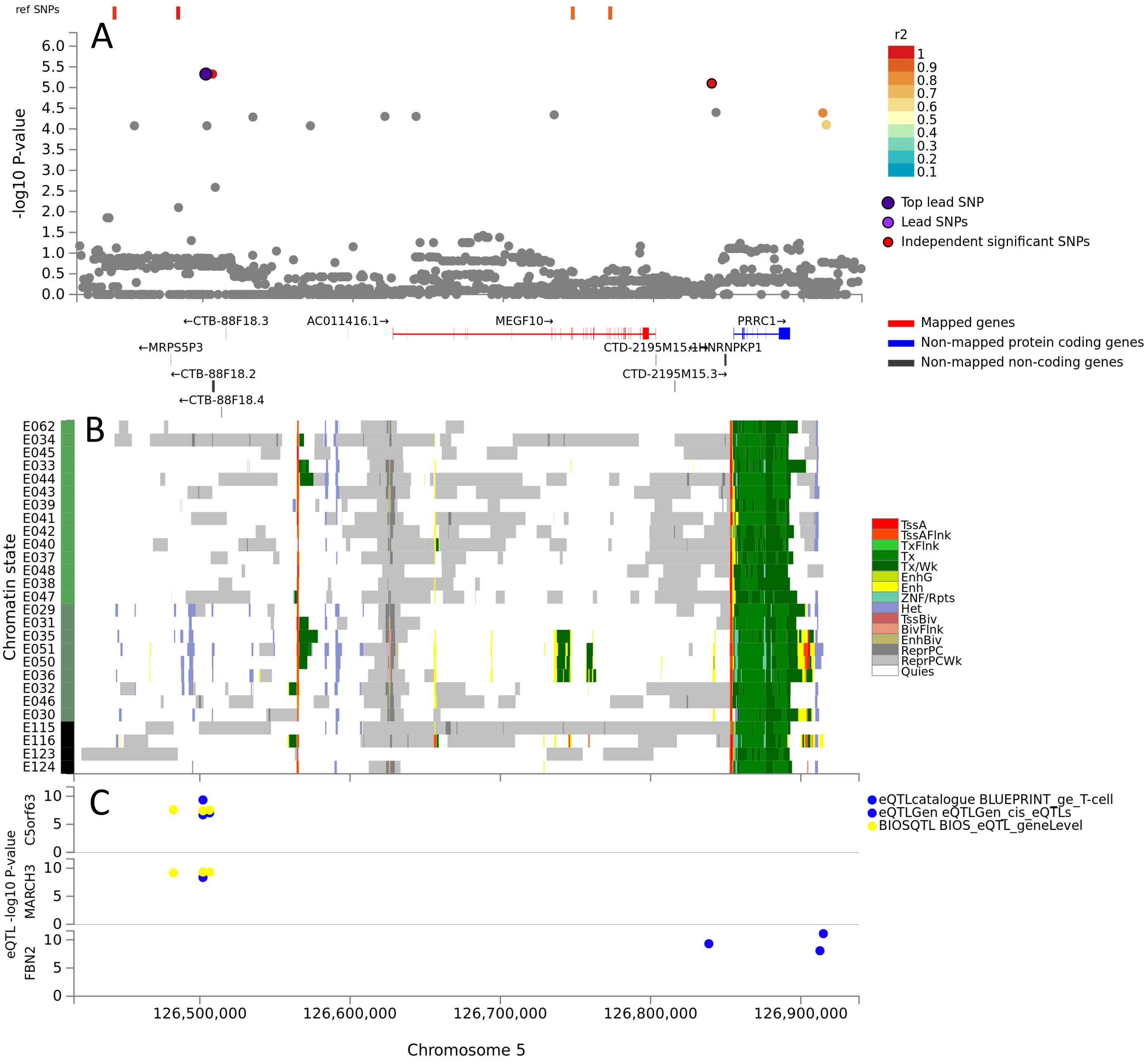

Figure S8. Results of positional, chromatin interaction, and eQTL activity mapping in FUMA for genomic locus 8 (*CTB-88F18.2*). (A) Maps the top lead SNV, and SNVs in LD with it according to the r^2 colour-coded key, across the three genes. There is one additional independent significant SNV. (B) Chromatin-15 states colour coded for transcriptional/enhancer activity as shown in the key. Y-axis colour coding relates to cell/tissue types in which chromatin interaction was mapped. (C) eQTL activity that relate to the top lead and independent SNVs for *MARCH3/C5orf63 and FBN2* genes (Y-axis), respectively, in different cells/tissues from public domain databases as shown in the key. *MARCH3/C5orf63* lie proximal to *CTB-88F18.2* and *MEGF10* on chromosome 5q, while *FBN2* lies distal (see Figure S11). Full explanation of keys provided on introductory page for supplementary figures.

Supplementary Figure 9

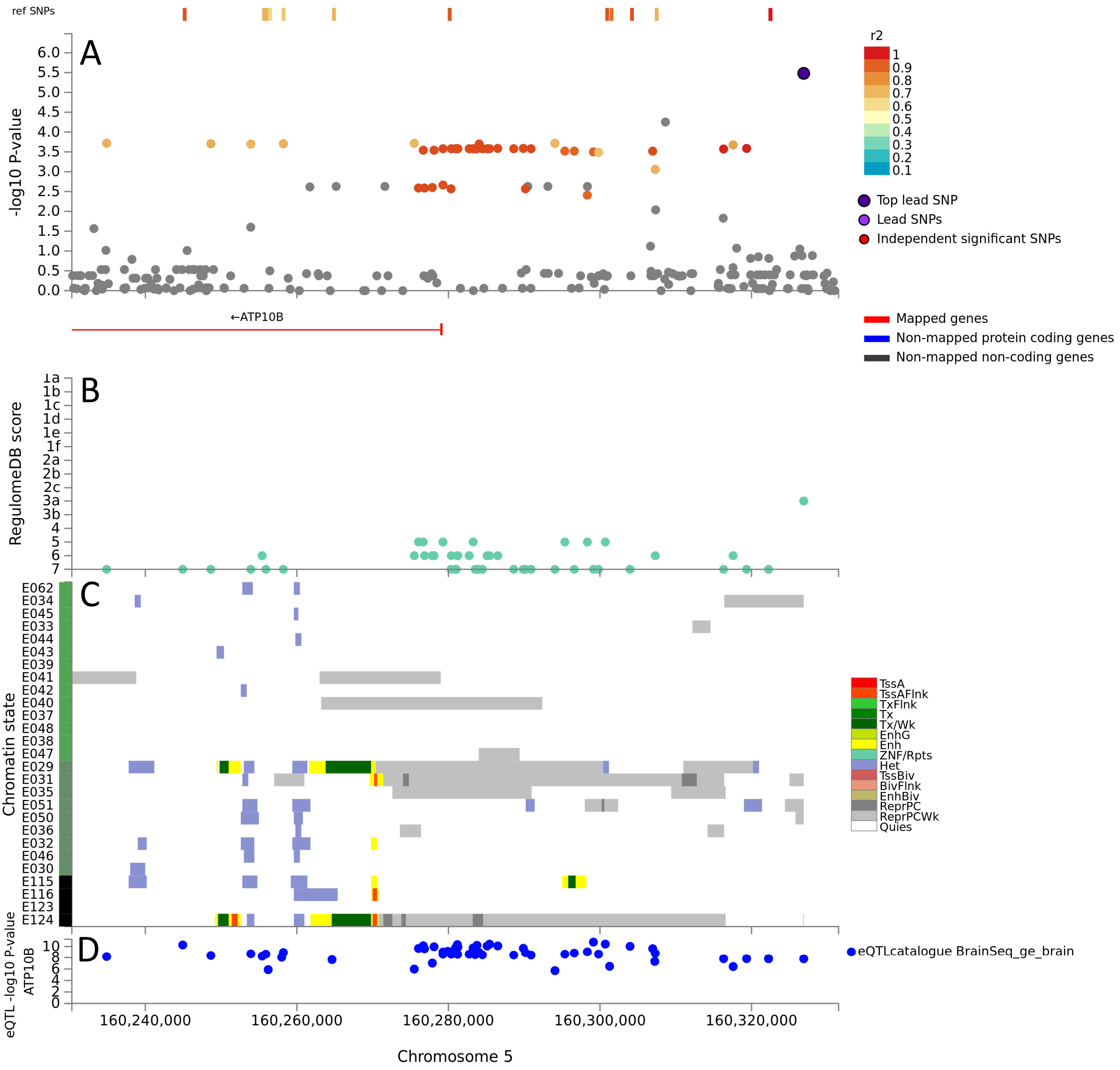

Figure S9. Results of positional, Regulome DB, chromatin interaction, and eQTL activity mapping in FUMA for genomic locus 12 (*ATP10B*). (A) Maps the top lead SNV, and SNVs in LD with it according to the r^2 colour-coded key, across the three genes. There were no additional independent significant SNVs. (B) Maps eQTL according to Regulome DB scores. (C) Chromatin-15 states colour coded for transcriptional/enhancer activity as shown in the key. Y-axis colour coding relates to cell/tissue types in which chromatin interaction was mapped. (D) eQTL activity for the *ATP10B* gene (Y-axis) in brain from public domain databases as shown in the key. Full explanation of keys provided on introductory page for supplementary figures.

Supplementary Figure 10

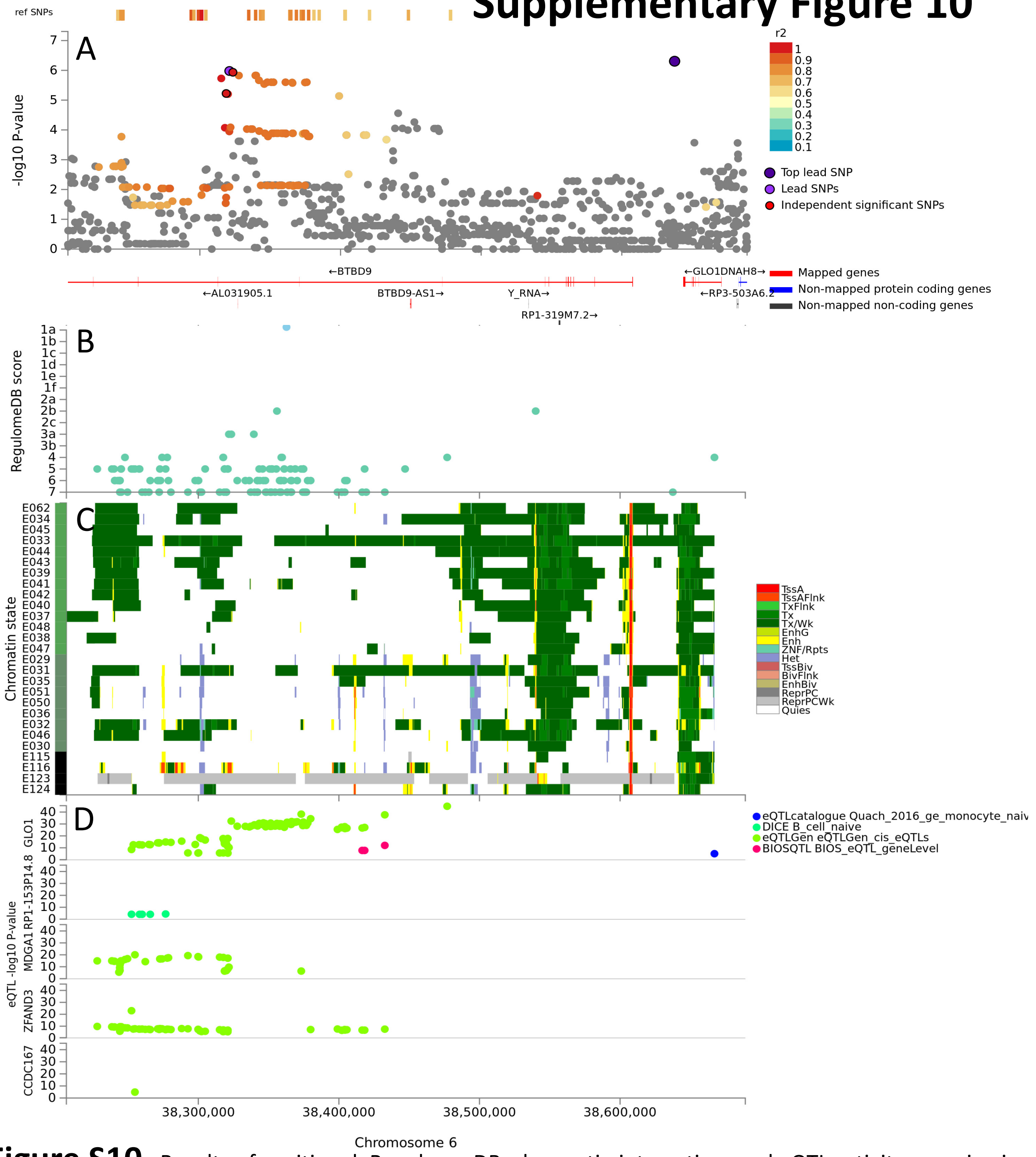

Figure S10. Results of positional, Regulome DB, chromatin interaction, and eQTL activity mapping in FUMA for genomic locus 14 (*GLO1*/*BTBD9*). (A) Maps the top lead SNV, and SNVs in LD with it according to the r^2 colour-coded key, across the region. There was one additional independent significant SNV. (B) Maps eQTL according to Regulome DB scores. (C) Chromatin-15 states colour coded for transcriptional/enhancer activity as shown in the key. Y-axis colour coding relates to cell/tissue types in which chromatin interaction was mapped. (D) eQTL activity for *GLO1* and other genes (Y-axis) in different cells/tissues from public domain databases as shown in the key. Note, there were no eQTL for *BTBD9*, and SNVs in LD with the independent significant SNV within *BTBD9* acted as strong cis-eQTL for *GLO1* expression. Full explanation of keys provided on introductory page for supplementary figures.

Supplementary Figure 11

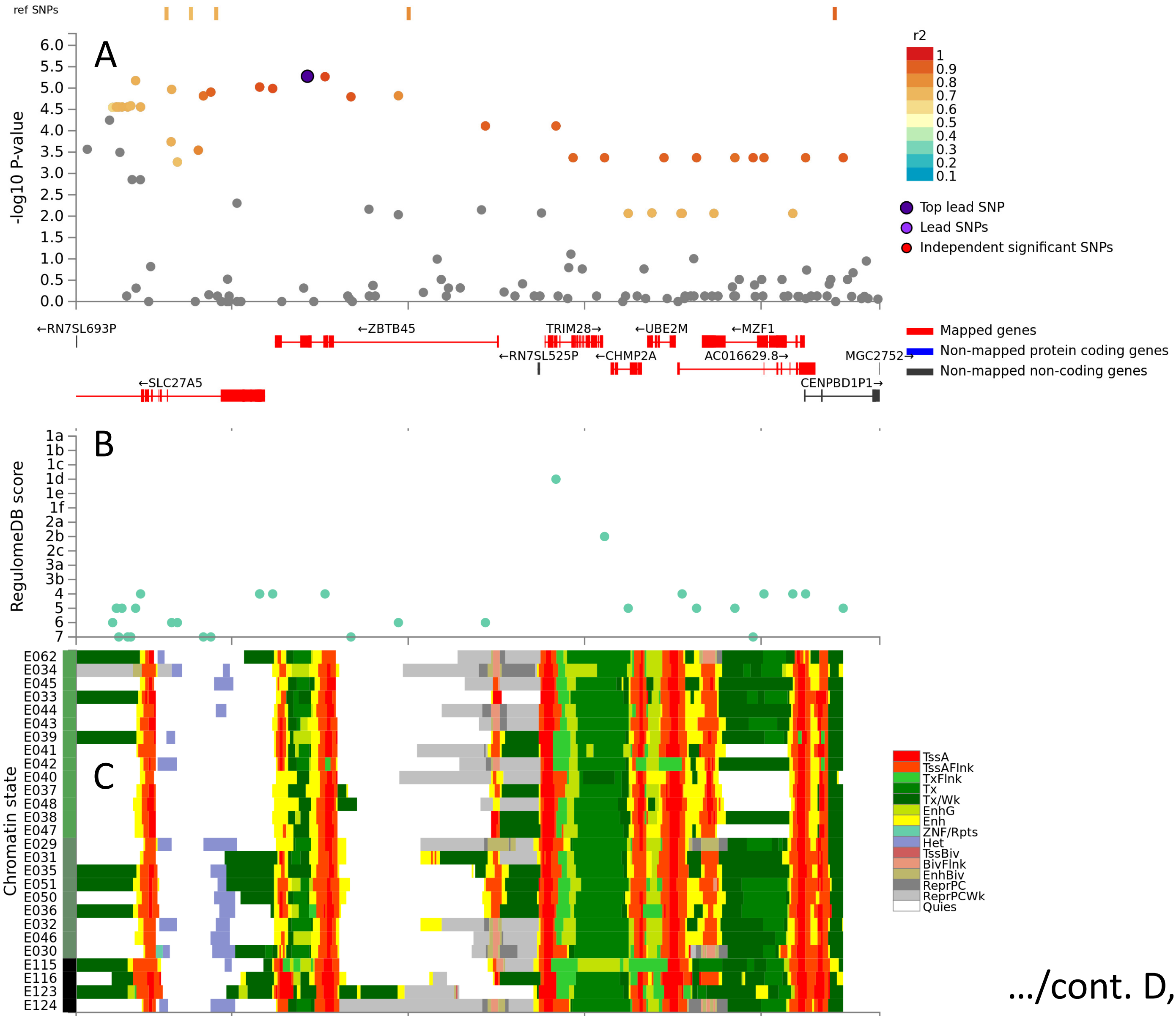

…/cont. D,E

Figure S11. Results of positional, Regulome DB, chromatin interaction, and eQTL activity mapping in FUMA for genomic locus 17 (*ZBTB45*). (A) Maps the top lead SNV, and SNVs in LD with it according to the r^2 colour-coded key, across the region. There was one additional independent significant SNV. (B) Maps eQTL according to Regulome DB scores. (C) Chromatin-15 states colour coded for transcriptional/enhancer activity as shown in the key. Y-axis colour coding relates to cell/tissue types in which chromatin interaction was mapped. (D) **see the following page** eQTL activity for *numerous* genes (Y-axis) in different cells/tissues from public domain databases as shown in the key (note there were no eQTL for *ZBTB45*). (E) Notes (taken from NCBI) on the many genes for which SNVs act as eQTL in this gene-dense and transcriptionally active region, making it difficult to identify a candidate OM risk gene in the region. Full explanation of keys provided on introductory page for supplementary figures.

Supplementary Figure 11 - continued

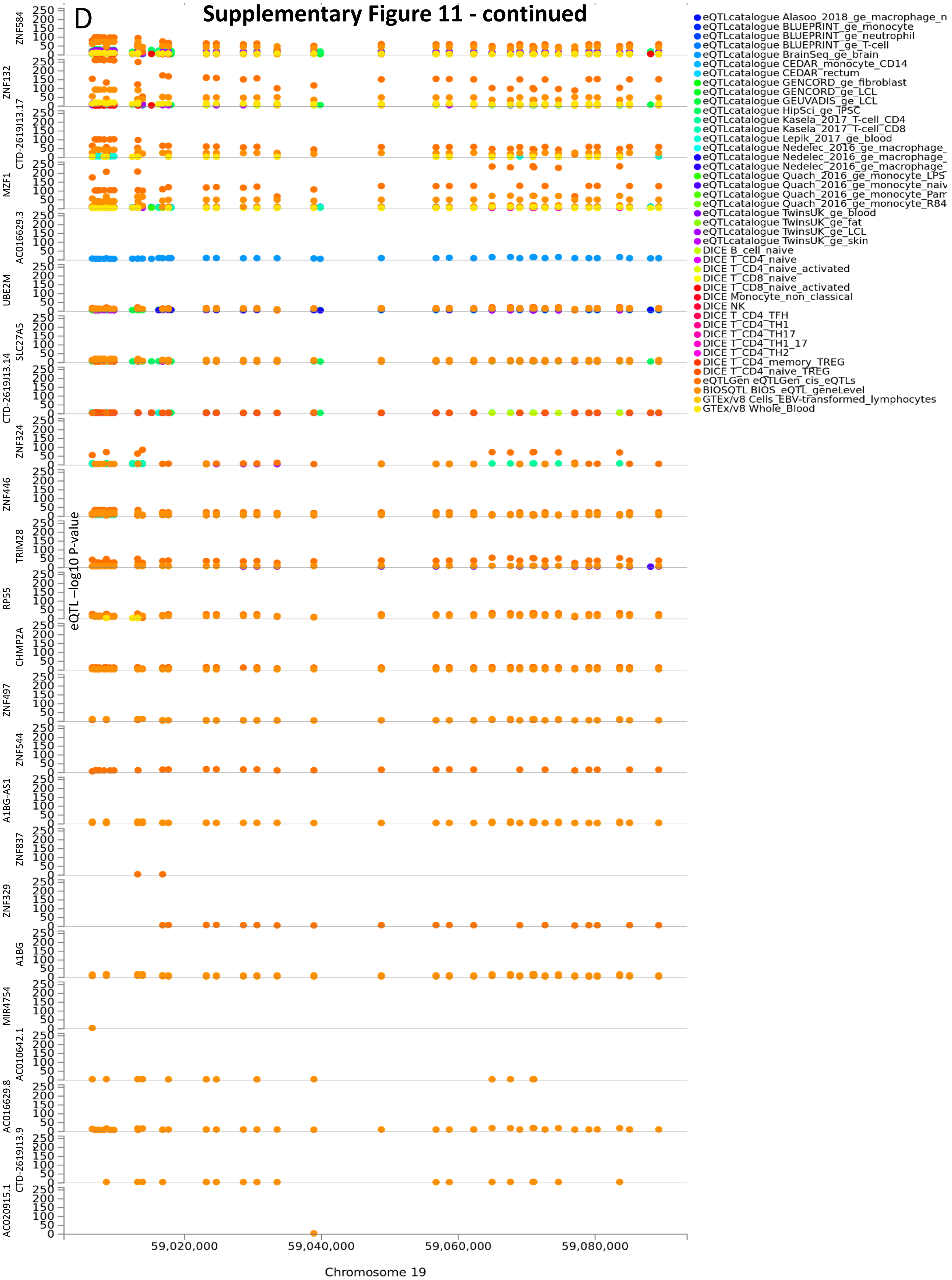

E

Supplementary Figure 11 - continued

- ZNF584, zinc finger protein 584, Ubiquitous expression in brain (RPKM 2.4), skin (RPKM 2.3) and 25 other tissues
- ZNF132, zinc finger protein 132, Ubiquitous expression in thyroid (RPKM 3.7), ovary (RPKM 2.5) and 25 other tissues
- CTD-2619J13.17, not found in NCBI gene or pubmed, eQTL for expression in blood/whole blood.
- MZF1, myeloid zinc finger 1, Ubiquitous expression in spleen (RPKM 4.6), prostate (RPKM 4.2) and 25 other tissues. 43eQTLs for eQTLcat monocyte CD14, 21 for eQTLcat rectum, 42 for eQTLcat T-cell_CD4 (10 shared with ZNF324), 28 for eQTLcat T-cell_CD8, 3 for eQTLcat monocyte LPS, 38 for eQTLcat blood (8 shared with ZNF132 and/or ZNF584)
- AC016629.3, LncRNA, overlapping ZNF8 sequence, 44 eQTLs for eQTLcat brain only.
- UBE2M, ubiquitin conjugating enzyme E2 M, The modification of proteins with ubiquitin is an important cellular mechanism for targeting abnormal or short-lived proteins for degradation. The encoded protein is linked with a ubiquitin-like protein, NEDD8, which can be conjugated to cellular proteins, such as Cdc53/culin. Ubiquitous expression in brain (RPKM 24.7), testis (RPKM 23.2) and 25 other tissues
- SLC27A5, solute carrier family 27 member 5, The protein encoded by this gene is an isozyme of very long-chain acyl-CoA synthetase (VLCS). It is capable of activating very long-chain fatty-acids containing 24- and 26-carbons. It is expressed in liver and associated with endoplasmic reticulum but not with peroxisomes. Its primary role is in fatty acid elongation or complex lipid synthesis rather than in degradation. Biased expression in liver (RPKM 126.6) and testis (RPKM 2.0)
- CTD-2619J13.14, not found in NCBI gene or pubmed, eQTL for expression in blood, and in DICE in all types of T cells and naïve B cells.
- ZNF324, zinc finger protein 324, Ubiquitous expression in brain (RPKM 2.5), ovary (RPKM 2.4) and 25 other tissues
- ZNF446, zinc finger protein 446, Ubiquitous expression in testis (RPKM 4.7), ovary (RPKM 4.0) and 25 other tissues
- TRIM28, tripartite motif containing 28, The protein mediates transcriptional control by interaction with the Kruppel-associated box repression domain found in many transcription factors. The protein localizes to the nucleus and is thought to associate with specific chromatin regions. The protein is a member of the tripartite motif family. This tripartite motif includes three zinc-binding domains, a RING, a B-box type 1 and a B-box type 2, and a coiled-coil region. Ubiquitous expression in ovary (RPKM 89.1), testis (RPKM 80.5) and 25 other tissues
- RP55, no information found.
- CHMP2A, charged multivesicular body protein 2A, CHMP2A belongs to the chromatin-modifying protein/charged multivesicular body protein (CHMP) family. These proteins are components of ESCRT-III (endosomal sorting complex required for transport III), a complex involved in degradation of surface receptor proteins and formation of endocytic multivesicular bodies (MVBs). Ubiquitous expression in colon (RPKM 34.2), kidney (RPKM 32.8) and 25 other tissues.
- ZNF544, zinc finger protein 544, Ubiquitous expression in thyroid (RPKM 3.1), testis (RPKM 3.0) and 25 other tissues
- ZNF497, zinc finger protein 497, Ubiquitous expression in brain (RPKM 1.3), endometrium (RPKM 1.2) and 25 other tissues
- A1BG-AS1, A1BG antisense RNA 1, Biased expression in liver (RPKM 106.5) and spleen (RPKM 1.9). Note: A1BG is below.
- ZNF837, zinc finger protein 837, Low expression observed in reference dataset.
- ZNF329, zinc finger protein 329, Ubiquitous expression in testis (RPKM 4.3), thyroid (RPKM 3.5) and 25 other tissues.
- A1BG, alpha-1-B glycoprotein, The protein encoded by this gene is a plasma glycoprotein of unknown function. The protein shows sequence similarity to the variable regions of some immunoglobulin supergene family member proteins. (No RefSeq expression data given).
- MIR4754, microRNA 4754, microRNAs (miRNAs) are short (20-24 nt) non-coding RNAs that are involved in post-transcriptional regulation of gene expression in multicellular organisms by affecting both the stability and translation of mRNAs. miRNAs are transcribed by RNA polymerase II as part of capped and polyadenylated primary transcripts (pri-miRNAs) that can be either protein-coding or non-coding.
- AC010642.1, part of complete sequence of Chr 19. No results pubmed.
- AC016629.8, part of complete sequence of Chr 19. No results pubmed.
- CTD-2619J13.9, not found in NCBI gene or pubmed, eQTL for BIOSQTL gene-level.
- AC020915.1, LncRNA, part of complete sequence of Chr 19. Within ZNF544/ZNF8 region. 1 eQTL only for BIOSQTL gene-level.

Supplementary Figure 12

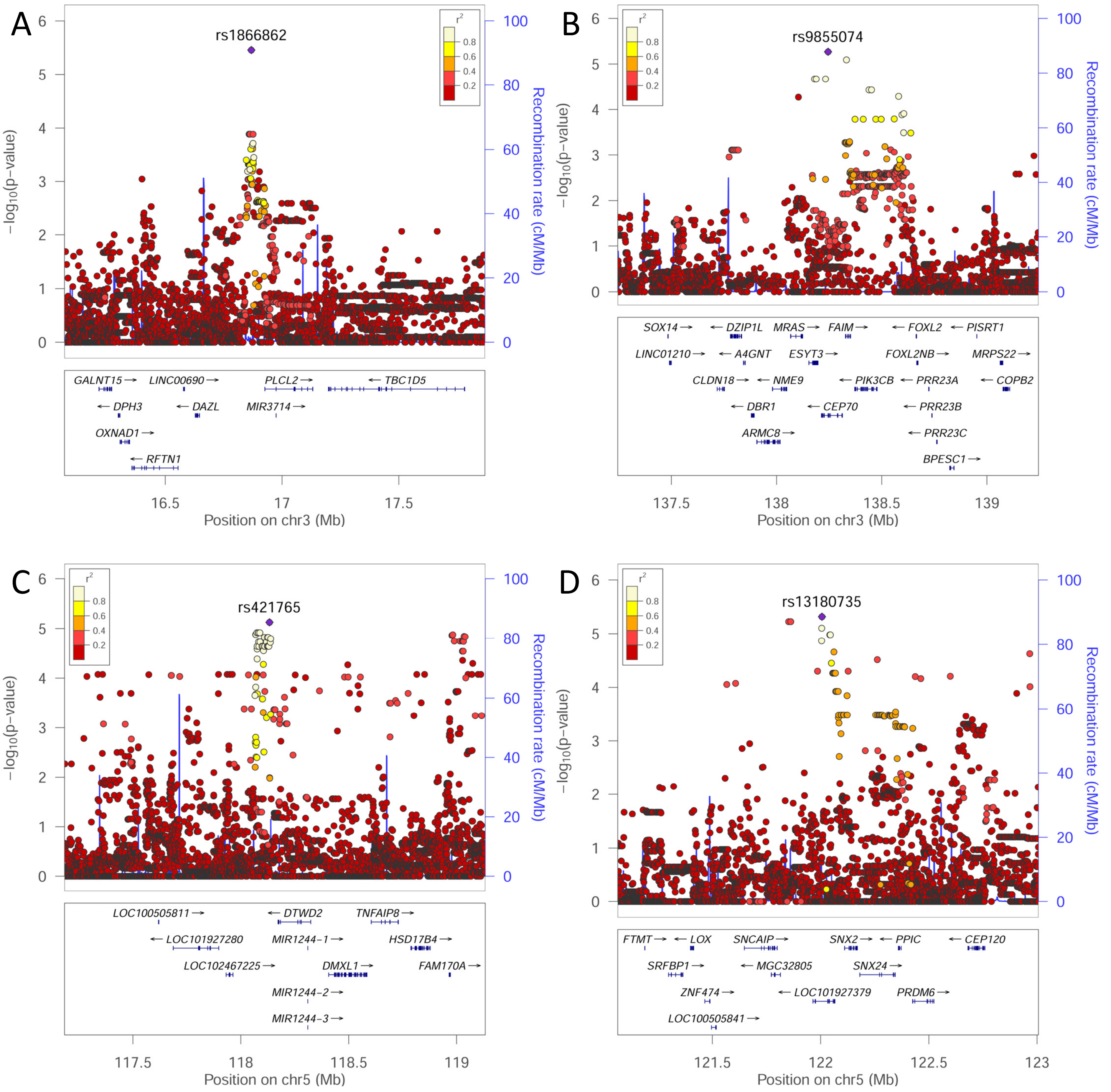

Figure S12. Regional association plots (LocusZoom[1]) of the signal for CSOM *per se* GWAS association in the regions of genomic loci (A to H) 1, 2, 4, 5, 8, 12, 14, 17 as determined using FUMA (see main Table 1). The $-\log_{10}$ P-values are shown on the left Y-axis of each plot. Dots representing individual SNVs are color coded (see key) based on their population-specific linkage disequilibrium r^2 with the top SNV (annotated by rs ID) in the region. The right Y-axis is for recombination rate (blue line), based on HapMap data. The bottom section of each plot shows the positions of genes across the region.

…/cont. E-H

Supplementary Figure 12 cont.

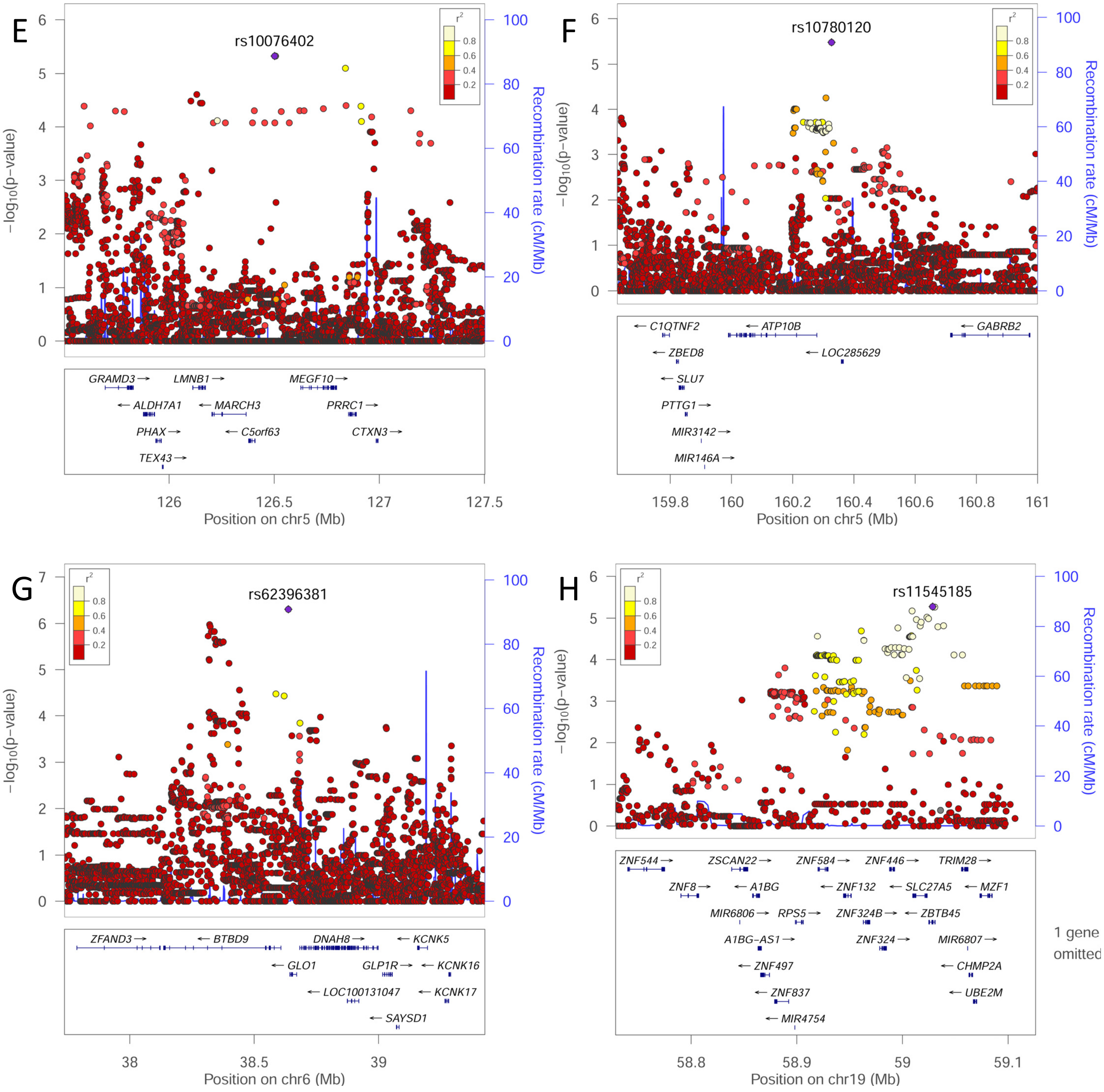

Figure S12 cont. Reference [1] Pruim RJ, Welch RP, Sanna S, *et al.* LocusZoom: regional visualization of genome-wide association scan results. Bioinformatics 2010; 26:2336-7.

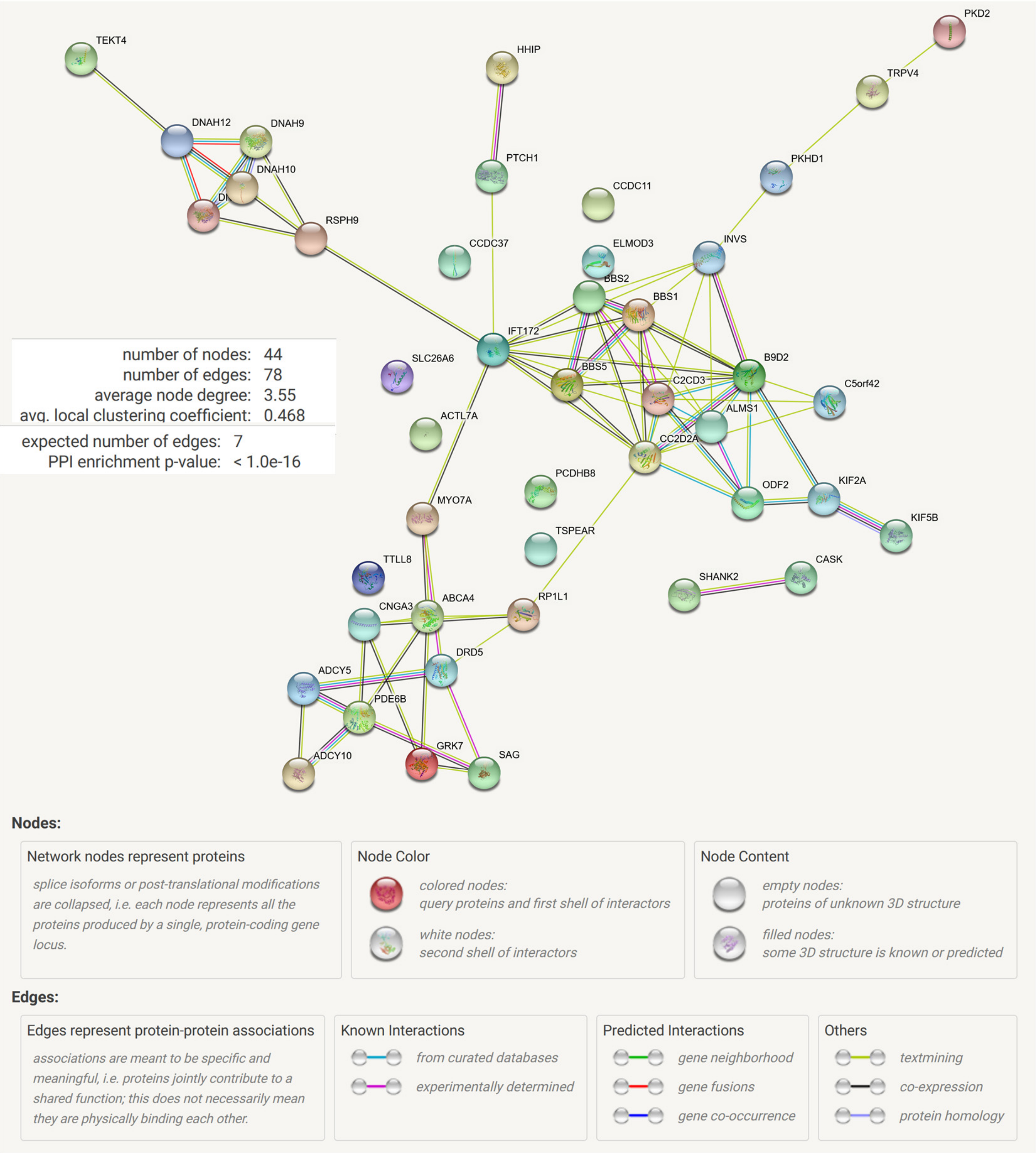

Figure S13. STRING interaction plot for the 44 genes with putative deleterious variants (CADD-scaled core ≥15) that Enirchr determined overlapped significantly ($P=1.16 \times 10^{-4}$; $P_{adjusted}=0.011$) with the 482 genes associated with "cilium" in the Jenson Compartments database (see Supplementary Table 7).
