# Supplementary material for: Common and Rare Genetic Variants That Could Contribute to Severe Otitis Media in an Australian Aboriginal Population
Source: Clin Infect Dis. 2021 Mar 9;73(10):1860–70. doi: 10.1093/cid/ciab216 (PMC8599203; doi:10.1093/cid/ciab216)
Supplement: ciab216_suppl_Supplementary_Table_S2 [file ciab216_suppl_supplementary_table_s2.docx]

**Supplementary Table 2.** Demographic (age at time of sample collection/sex ratio) details for the 111 individuals with definitive OM phenotypes that were used in the GWAS. A subset of the severe (N=15) and mild OM (single isolated episode of acute OM only) (N=9) groups were used in the exome-based extreme phenotype analyses.

|  | Severe | Intermediate | Mild OM | No OM |
| --- | --- | --- | --- | --- |
| Average age at collection | 7.29 | 5.48 | 12.11 | 14.05 |
| Standard Deviation | 3.81 | 4.34 | 6.66 | 6.25 |
| Age range | 1 to 15 | 1 to 17 | 1 to 26 | 2 to 27 |
| N | 21 | 21 | 28 | 41 |
| Sex ratio (M:F) | 9:12 | 13:8 | 17:11 | 17:24 |
